# Mass spectrometry characterization of light chain fragmentation sites in cardiac AL amyloidosis: insights into the timing of proteolysis

**DOI:** 10.1074/jbc.RA120.013461

**Published:** 2021-01-13

**Authors:** Francesca Lavatelli, Giulia Mazzini, Stefano Ricagno, Federica Iavarone, Paola Rognoni, Paolo Milani, Mario Nuvolone, Paolo Swuec, Serena Caminito, Masayoshi Tasaki, Antonio Chaves-Sanjuan, Andrea Urbani, Giampaolo Merlini, Giovanni Palladini

**Affiliations:** 1 Amyloidosis Research and Treatment Center, Fondazione IRCCS Policlinico San Matteo and University of Pavia, Pavia, Italy; 2 Department of Biosciences, Università degli Studi di Milano, Milan, Italy; 3 Department of Basic Biotechnological Sciences, Intensivological and Perioperative Clinics, Faculty of Medicine, Università Cattolica del Sacro Cuore, Rome, Italy; 4 Clinical Chemistry, Biochemistry and Molecular Biology Clinic, Fondazione Policlinico Agostino Gemelli IRCCS, Rome, Italy; 5 Cryo-Electron Microscopy Facility, Human Technopole, Milan, Italy; 6 Department of Morphological and Physiological Sciences, Graduate School of Health Sciences, Kumamoto University, Kumamoto, Japan; 7 Department of Neurology, Graduate School of Medical Sciences, Kumamoto University, Kumamoto, Japan

**Keywords:** amyloid fibrils, proteolysis, cardiomyopathy, amyloid, protein aggregation, protein conformation, protein structure, proteomics, mass spectrometry (MS), structural biology, fibril

## Abstract

Amyloid fibrils are polymeric structures originating from aggregation of misfolded proteins. *In vivo*, proteolysis may modulate amyloidogenesis and fibril stability. In light chain (AL) amyloidosis, fragmented light chains (LCs) are abundant components of amyloid deposits; however, site and timing of proteolysis are debated. Identification of the N and C termini of LC fragments is instrumental to understanding involved processes and enzymes. We investigated the N and C terminome of the LC proteoforms in fibrils extracted from the hearts of two AL cardiomyopathy patients, using a proteomic approach based on derivatization of N- and C-terminal residues, followed by mapping of fragmentation sites on the structures of native and fibrillar relevant LCs. We provide the first high-specificity map of proteolytic cleavages in natural AL amyloid. Proteolysis occurs both on the LC variable and constant domains, generating a complex fragmentation pattern. The structural analysis indicates extensive remodeling by multiple proteases, largely taking place on poorly folded regions of the fibril surfaces. This study adds novel important knowledge on amyloid LC processing: although our data do not exclude that proteolysis of native LC dimers may destabilize their structure and favor fibril formation, the data show that LC deposition largely precedes the proteolytic events documentable in mature AL fibrils.

Amyloid fibrils are polymeric structures originating from the aggregation of misfolded proteins. In humans, at least 36 distinct autologous proteins can cause amyloid diseases (1). The mechanisms leading to fibrillogenesis *in vivo* are still largely unknown; however, interaction with tissue components and proteolytic processing by circulating or tissue proteases are thought to play an important role in modulating protein stability and aggregation. Amyloid deposits are often composed of fragments of the precursor protein (2, 3, 4, 5, 6, 7, 8, 9). Proteolysis has a plausible pathogenic role *in vivo* in amyloid diseases such as Alzheimer's disease (10) and systemic transthyretin (ATTR) amyloidosis (11, 12), whereas its causative role in other forms is still debated (6).

The most prevalent acquired systemic amyloidosis in Western countries is light chain amyloidosis (AL), caused by aggregation and deposition of monoclonal immunoglobulin free light chains (LCs) that are produced in excess by a bone marrow plasma cell clone. Light chains are ∼22–23-kDa proteins composed of a variable region, a joining segment, and a constant region. A complex mechanism of germline gene recombination and somatic hypermutation translates into high variability among LCs, especially at the level of VL, so that the sequence of each patient's monoclonal protein is virtually unique (13).

The *ex vivo* amyloid deposits in AL amyloidosis are composed of a heterogeneous mixture of LC proteoforms, with a complex ensemble of fragments (6, 7). The presence of the entire variable domain (VL) in the most abundant fragments (7) and the increased amyloidogenic potential of the isolated VL compared with the full-length LCs lead to postulate that VL may have a crucial importance in amyloid fibril formation (14, 15, 16). This hypothesis has been recently supported by two fibril structures (17, 18), determined by cryo-EM, in which VL was shown to constitute the rigid core of the fibril, which is largely resistant to limited proteolysis. These observations are in favor of the hypothesis that proteolysis, with release of free VL, may occur prior to LC deposition. On the other hand, however, a definite, single LC amyloidogenic fragment, whose greater abundance could support a nucleation/seeding effect, has not yet been identified in AL fibrils extracted under rigorous protease inhibition. In addition, established evidence, largely derived from proteomic studies, indicates that full-length LCs are also invariable constituents of the deposits (7, 19, 20) and fragments containing exclusively portions of the light chain constant domain (CL) have been detected (6, 21). These considerations, and the evidence that AL fibrils can be effectively degraded *in vitro* by tissue proteases (22, 23), support the alternative hypothesis that proteolysis may largely occur after fibril deposition.

Defining the site and timing of the proteolytic events in AL *in vivo* has two important implications: a better understanding of amyloid formation in the natural environment and a therapeutic perspective to prevent destabilizing proteolysis (24) or to potentiate the tissue defenses that degrade harmful aggregates (22).

Identification of the N and C termini of the LC fragments is instrumental to understand the enzymes and processes involved in cleavage. This task is especially challenging in AL given the complexity of the fragmentation pattern. Past studies, based on biochemical investigations, amino acid analysis, and protein sequencing, detected fragmentation points that, across the various LCs, were located in the N-terminal part of the constant region (around amino acids 110, 130, and 150) (21, 25). These studies, however, could not map the primary sequences of all the fragments but rather of the most abundant ones. More recently, the advent of MS-based proteomics has deeply changed the analytical sensitivity, allowing the assessment of primary sequence and post-translational modifications of protein isoforms in complex protein mixtures. Two-dimensional PAGE–based studies (7, 17, 20), for example, allowed for dissecting LC proteoforms (fragments and pI isoforms) with very high resolving power. MS analysis of proteins from amyloid deposits, moreover, allowed dissecting a heterogeneous series of C-terminal transthyretin fragments in ATTRwt amyloidosis (5). However, challenges still often exist in identifying proteolytic sites using standard bottom-up proteomics approaches. Several factors, such as scarce control of trypsin digestion and of peptide extraction from gels, in-source peptide fragmentation, and possible lack of detection of some peptides during MS analysis, translate into uncertainty regarding the exact protein termini. Labeling strategies that introduce MS-detectable stable chemical modifications on the C- and N-terminal amino acids are required to increase confidence in assigning terminal residues.

The purpose of this study was to fill the gap of knowledge regarding the fragmentation sites in amyloid light chains by obtaining a comprehensive map of the N- and C-terminal amino acids of all the LC proteoforms from AL deposits. To this aim, we implemented a dedicated proteomic approach based on chemical derivatization of the free terminal carboxyl and amino groups of the LC, followed by MS-based identification of the labeled residues. We applied this approach to the analysis of amyloid LC extracted from the hearts of two patients with AL λ cardiomyopathy; the identified cleavage sites were then mapped on the proteins' native and fibrillar structures to gain information on the accessibility to proteolysis in either conformation.

## Results

**Figure 1:**
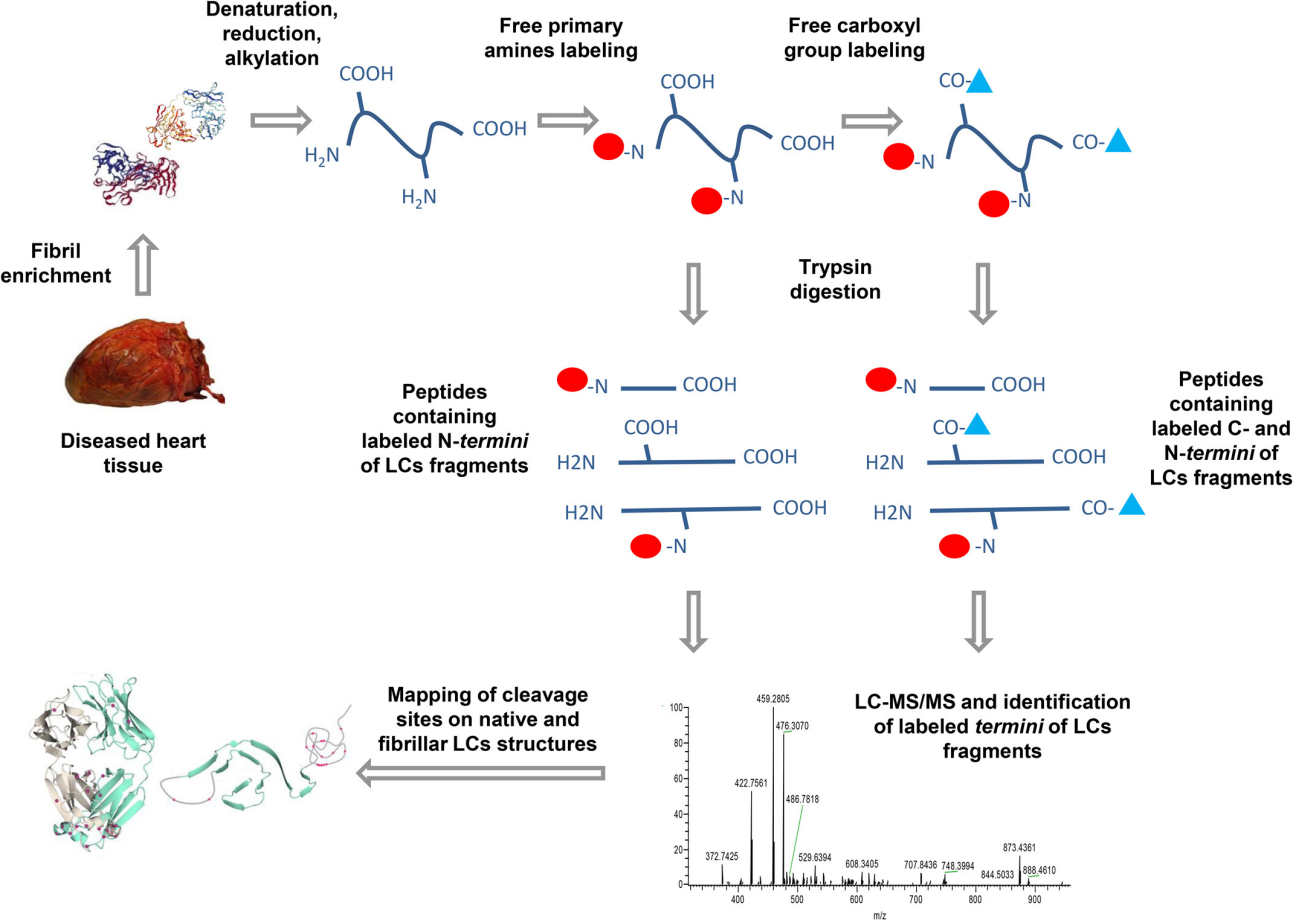
Graphic scheme of the workflow for the analysis of the N- and C-terminal residues of the light chain proteoforms extracted from AL amyloid deposits in heart tissue.

We have characterized, using MS coupled with derivatization of free N-terminal amino groups and free C-terminal carboxyl groups, the termini of the LC proteoforms deposited as amyloid fibrils in the hearts of two patients affected by AL λ amyloidosis, containing deposits of LCs named AL-H7 and AL-55, as previously reported (17, 26). The proteomic workflow is summarized in Fig. 1. Fibrils were extracted from the diseased tissues using a gentle procedure and then solubilized, followed by gel electrophoresis, or in-solution derivatization and LC–MS/MS. With the extensive use of protease inhibitors and in the absence of enzymatic treatments, heart-derived amyloid fibrils are tightly entangled to collagen and the yield of water-extracted material is low, because most fibrils remain in the insoluble pellet (data not shown). Because of this consideration, we opted for the analysis of the whole pellet remaining after repeated washes with Tris EDTA buffer without proceeding to extensive water extraction (27). In fact, during homogenization in Tris EDTA buffer, blood and tissue proteins soluble in saline solutions are depleted and fibrils are enriched.

### Gel electrophoresis characterization of the LCs from amyloid deposits

**Figure 2:**
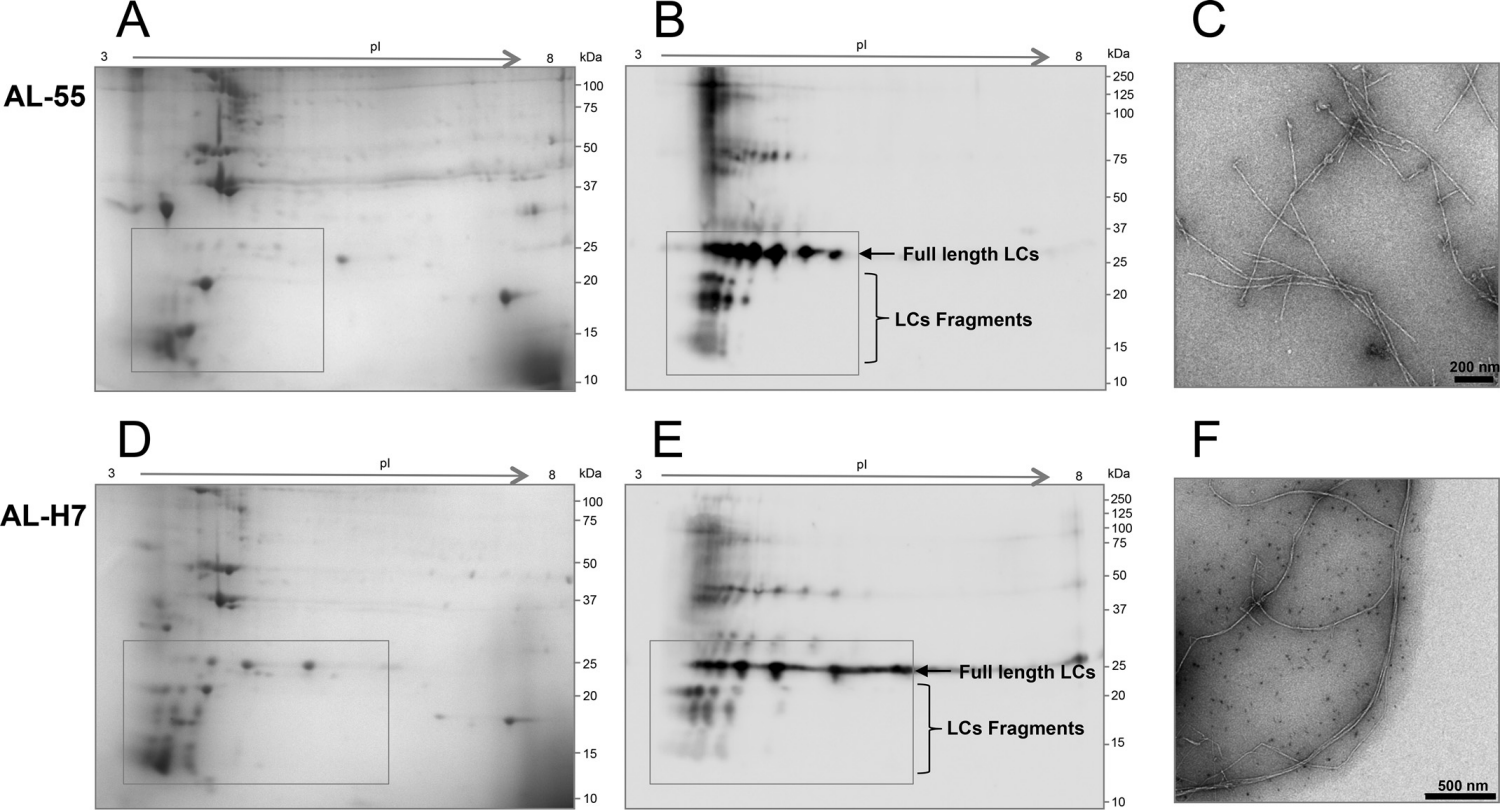
**Two-dimensional PAGE analysis of protein pellets after 10 cycles of heart homogenization and washing in Tris EDTA buffer.***A* and *B*, respectively, Coomassie-stained gel and Western blotting referring to AL-55. *C*, cryo-EM image of AL-55 fibrils, extracted from heart and analyzed as described by Mangione *et al. D* and *E*, respectively, Coomassie-stained gel and Western blotting images referring to AL-H7. *F*, cryo-EM image of AL-H7 fibrils, extracted from heart and analyzed as described. *Boxed areas* in gels and blots (25 kDa to 15–12 kDa, approximately) correspond to regions of gel where the full-length LCs and visible fragments, in monomeric form, migrate. Higher molecular weight LC species are also visible, which may correspond to cross-linked or incompletely reduced species. The uncropped gel image corresponding to *panel A*, including the MW marker lane, is shown in Fig. S5.

The 2D electrophoresis and immunoblot study of the solubilized protein pellets are shown in Fig. 2 and Fig. S5. Light chains from fibrils constitute a relevant fraction of the proteins in the samples; however, other heart tissue proteins are present in the protein pellets. No blood-borne proteins were instead identified as significant components in either sample, with the exception of common constituents of amyloid deposits (apolipoprotein E, clusterin, serum amyloid P, and apolipoprotein A-IV) (28). As expected, Western blotting showed the presence, in both AL-55 and AL-H7 samples, of numerous λ LC species with molecular weight ranging between ∼25 and ∼15–12 kDa (Fig. 2). It should be noted, however, that the fragments identified by immunoblot may not represent the whole fragment population present in the fibrils, because short fragments containing exclusively the VL, or missing major portions of the CL, are not efficiently recognized by commercial antibodies, as previously noted (17). Significant amounts of LC spots consistent with the full-length protein were also detected in both samples. In particular, the fraction of full-length AL-55 observed in the present experiments is higher compared with the one reported in Swuec *et al.* (17) after overnight treatment of cardiac tissue with collagenase. This is likely because collagenase treatment, or lack of endogenous proteases inhibition, causes partial digestion of the outer, loosely packed region of the fibrillar assemblies largely constituted by the CL, whereas proteolysis does not significantly affect the tightly packed fibril core formed by the VL (17, 18).

### Identification of the N-terminal amino acids of the LC fragments

The N-terminal amino acids of the LC fragments were identified through derivatization of free primary amines by reductive dimethylation. The presence of a derivatized N-terminal amino group on a semitryptic peptide (double derivatization of the two free amino groups in case of lysine residues) indicates that the labeled amino acid is the N terminus of the corresponding fragment. The reaction was performed before *in vitro* trypsin proteolysis to allow distinguishing original N termini from peptide N termini newly generated after digestion. Upon LC–MS/MS analysis, 197 and 448 distinct proteins overall (corresponding to 5,207 and 9,161 peptides) with ≥2 unique peptides were identified in the AL-55 and AL-H7 samples, respectively. As stated above, with the exception of the two LCs these proteins correspond to tissue proteins remaining in the pellet after the washings and to the “amyloid proteomic signature” proteins (28). More than 99% of the detected proteins contained peptides with dimethylated N termini (Fig. S1) in canonical (*i.e.* occurring in first position or after a signal peptide) or in internal/noncanonical positions (29, 30).

**Figure 3:**
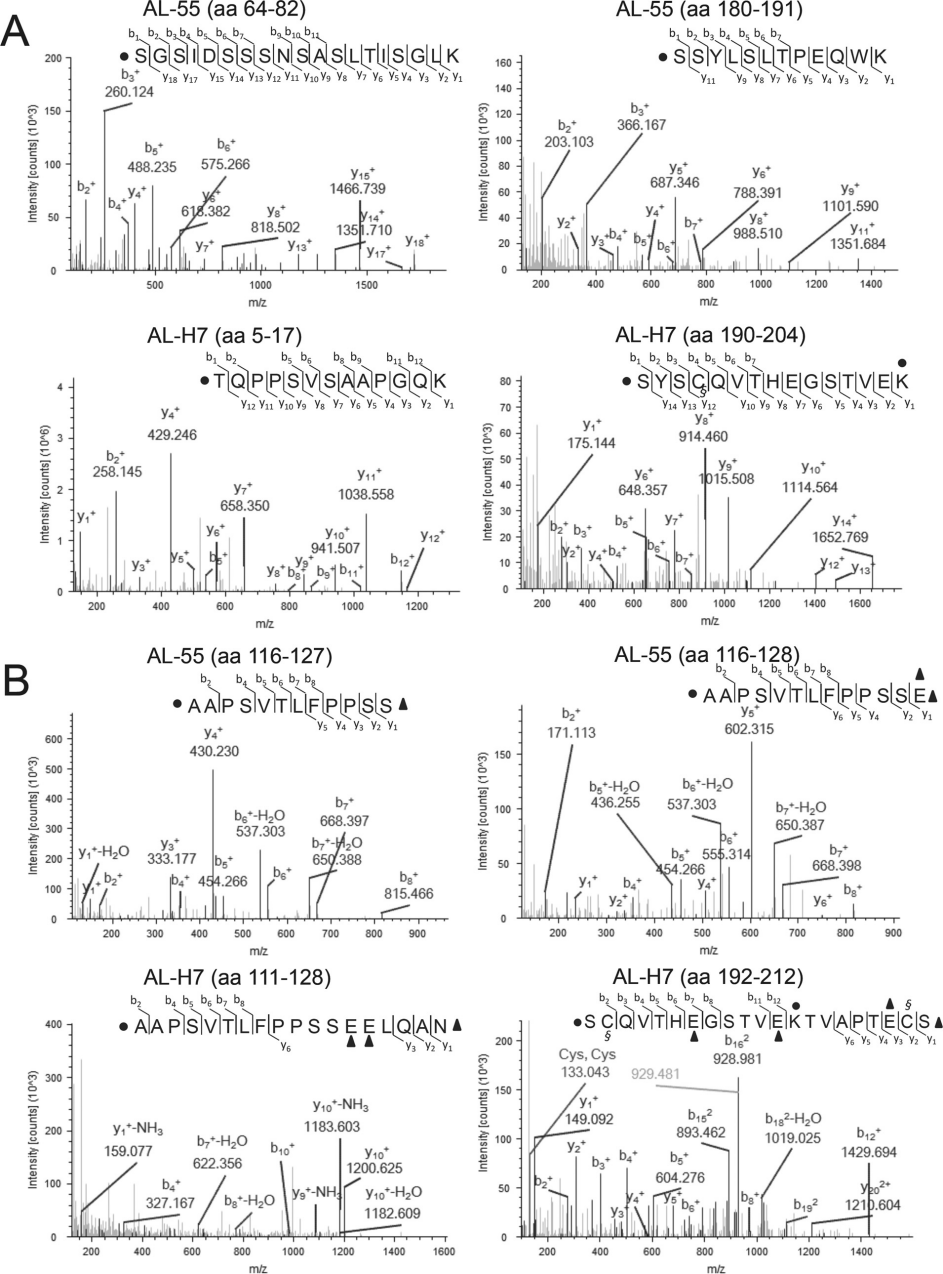
*A*, N-terminomics analysis: MS/MS spectra of AL-55 peptides 64–82, AL-55 peptides 180–191, AL-H7 peptides 5–17, and AL-H7 peptides 190–204. ● amino acid carrying dimethylation; § amino acid carrying carbamidomethylation. *B*, C-terminomics analysis: MS/MS spectra of AL-55 peptides 116–127, AL-55 peptides 116–128, AL-H7 peptides 111–128, and AL-H7 peptides 192–212. ● amino acid carrying dimethylation; § amino acid carrying carbamidomethylation; ▴ amino acid carrying ethanolamine. Mass measurement accuracy, calculated as the average Δ *m*/*z* (experimental-theoretical value) of all peptide spectrum matches attributed to labeled peptides of AL-55 and AL-H7, is 0.004 Da.

**Figure 4:**
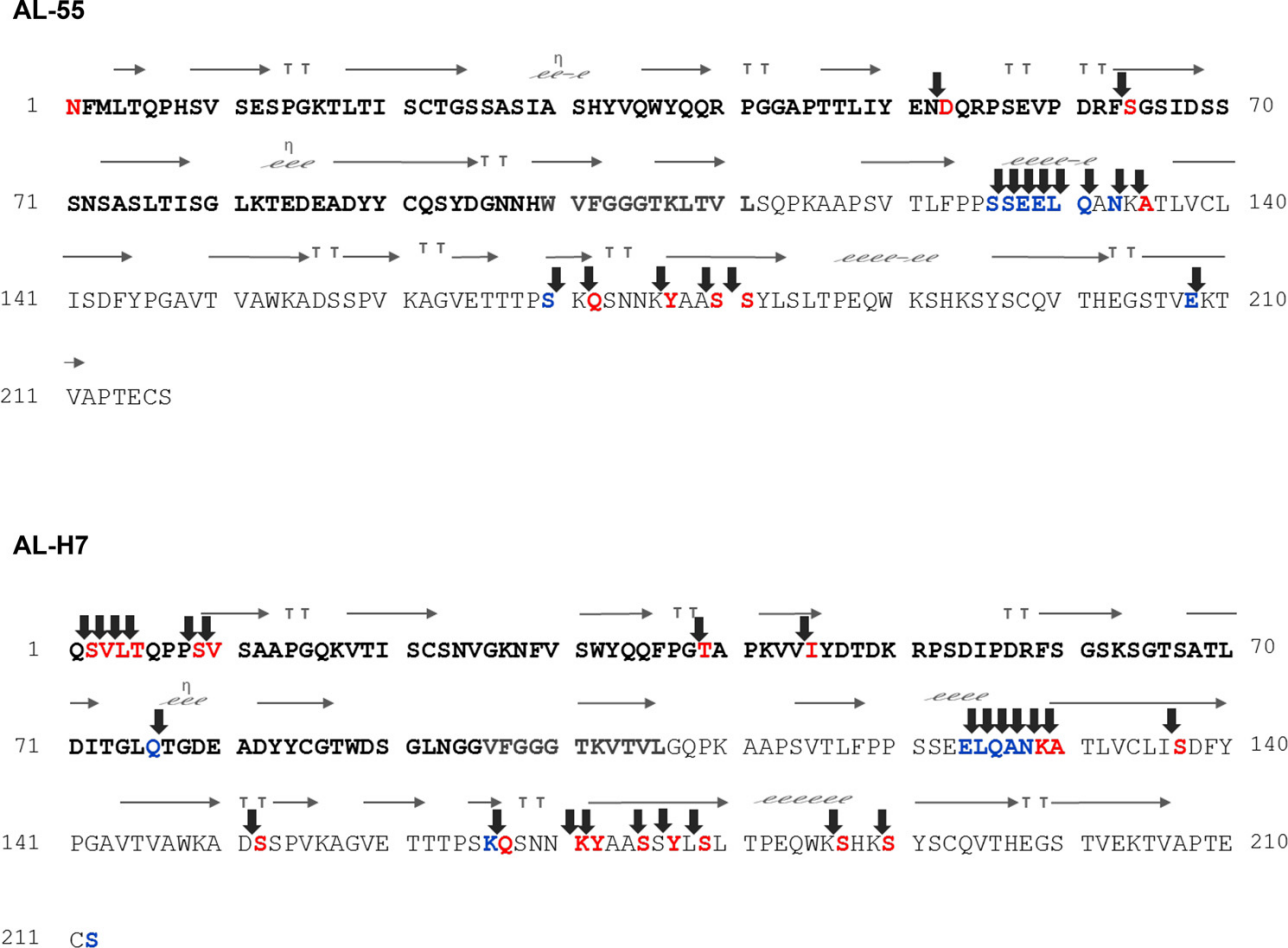
**Primary sequences of AL-55 (*top*) and of AL-H7 (*bottom*) together with the associated secondary structures.** The LCs' variable regions (aa 1–99 in AL55 and 1–94 in AL-H7) are indicated in *bold black*; the joining regions (aa 100–111 in AL-55 and 95–106 in AL-H7) are displayed in *bold gray*. Identified fragmentation sites (indicated by *arrows*) along the amino acid sequences. The residues identified as labeled on the carboxylic group are indicated in *blue* and correspond to the C terminus of the corresponding fragment. Conversely, the residues identified as labeled on the amino group are indicated in *red* and correspond to the N terminus of the corresponding fragment. Secondary structures were calculated by Endscript 2.0 (RRID:SCR_006587) using a model of AL-55 structure based on JTO crystal structure (PDB entry: 6MG4) and of AL-H7 crystal structure (PDB entry: 5MUH). On top of residues structured in β strands or in α helices, *arrows* or *helices* are shown, respectively. *TT* are β-turn, η left-handed α helices.

Focusing on AL-55 and AL-H7 LCs, sequence coverage was 100% for both proteins. Dimethylated N-terminal residues were, respectively, eight in AL-55 and 20 in AL-H7. The annotated spectra of labeled peptides are shown in Fig. 3*A* and in Fig. S2. In AL-55, three of the identified N termini were located in the variable domain (Asn^1^, *i.e.* N terminus of the full-length LC, Asp^53^, and Ser^64^) and five were located in the constant domain (Ala^135^, Gln^172^, Tyr^177^, Ser^180^, and Ser^181^) (Fig. 4, Fig. S2, Fig. S6, and Table S2). In AL-H7, eight dimethylated N termini were located in the VL (Ser^2^, Val^3^, Leu^4^, Thr^5^, Ser^9^, Val^10^, Thr^39^, and Ile^45^) and 12 were located in the CL (Lys^129^, Ala^130^, Ser^137^, Ser^152^, Gln^167^, Lys^171^, Tyr^172^, Ser^175^, Tyr^177^, Ser^179^, Ser^187^, and Ser^190^) (Fig. 4, Fig. S2, Fig. S6, and Table S2). The expected N terminus (Gln^1^) was only detected with pyroglutamate cyclization (data not shown). Compared with AL-55, more labeled positions were found in AL-H7, mostly located in the 10 N-terminal residues. Four of the labeled positions in the constant region (Ala^130^ in AL-H7, corresponding to Ala^135^ in AL-55; Gln^167^ in AL-H7, corresponding to Gln^172^ in AL-55; Ser^175^ in AL-H7, corresponding to Ser^180^ in AL-55; and Tyr^172^ in AL-H7, corresponding to Tyr^177^ in AL-55) are identical in the two LCs.

### Identification of the C-terminal amino acids of the LC fragments

To investigate the C-terminal amino acids of the LC fragments, free carboxyl groups were labeled with ethanolamine. The presence of a derivatized carboxyl group on the C terminus of a semitryptic peptide indicates that the labeled amino acid is the C terminus of the corresponding protein fragment (in case the C-terminal residue of a peptide was an aspartate or glutamate, only doubly derivatized peptides were considered as *bona fide* C termini). Unmodified internal peptides originated by trypsin cleavage were depleted by condensation with polyallylamine (PAA), aiming to reduce sample complexity and to improve detection of minor labeled species. However, to account for loss of potentially informative peptides, corresponding labeled samples without PAA treatment were also analyzed in parallel. Indeed, derivatization on side chains was not complete, so that some peptides labeled at the C terminus and not on the side chains may have been lost through PAA condensation. Moreover, we speculated that potential deamidation of asparagine and glutamine residues during processing (whose assessment was beyond the scope of this study) could in principle originate a novel carboxyl group that could also react with PAA. Therefore, all manually confirmed labeled peptides identified in either analysis were considered and included in the results described in the following paragraphs.

Overall, C-terminal labeling was less effective than N-terminal derivatization because of the lower chemical reactivity of the free carboxyl groups (31), and fewer derivatized C termini were detected compared with the N-terminal derivatization (Fig. S1).

Focusing on amyloid LCs, the derivatized canonical C terminus of the full-length sequence was only found in AL-H7 (Ser^212^), although the unlabeled C-terminal peptide was detected in both sequences. In AL-H7, additional labeled C-terminal amino acids were detected at one position (Gln^76^) in the VL and at six different positions (Glu^124^, Leu^125^, Gln^126^, Ala^127^, Asn^128^, and Lys^166^) in the CL (Fig. 4 and Fig. S6). In AL-55, labeled C-terminal amino acids were identified at nine additional positions along the sequence (Ser^126^, Ser^127^, Glu^128^, Glu^129^, Leu^130^, Gln^131^, Asn^133^, Ser^170^, and Glu^208^), all of them in the constant region (Fig. 4 and Fig. S6). Four of the labeled positions in the CL are identical in the two LCs (Glu^124^ in AL-H7, corresponding to Glu^129^ in AL-55; Leu^125^ in AL-H7, corresponding to Leu^130^ in AL-55; Gln^126^ in AL-H7, corresponding to Gln^131^ in AL-55; and Asn^128^ in AL-H7, corresponding to Asn^133^ in AL-55).

All these labeled residues can be confidently interpreted as C termini of the corresponding LC fragments. Most of these truncation sites are located in the N-terminal part of the CL and indicate trimming of the protein at this site. Fragments generated by cleavage in this region and encompassing the whole VL would have a molecular weight (MW) of ∼13–14 kDa, consistent with the results obtained by PAGE (Fig. 2 and Fig. S6). In AL-H7, two of the identified novel C termini were complementary to identified novel N termini (Asn^128^-Lys^129^ and Ser^166^-Gln^167^), indicating that both peptides generated by cleavage at these points are present in the sample. The paucity of complementary positions could be related to further degradation phenomena after initial cleavages but also to a lack of labeling of some positions, which is expected to be more significant in the case of the scarcely reactive C-terminal carboxyl groups (31).

The positions of all the identified fragmentation sites for each LC are graphically summarized in Fig. 4, in which the N-terminal residues of the corresponding fragments are colored in *red* and the C-terminal residues of the corresponding fragments are colored in *blue*.

### Mapping cleavage sites on native and fibrillar light chain structures

**Figure 5:**
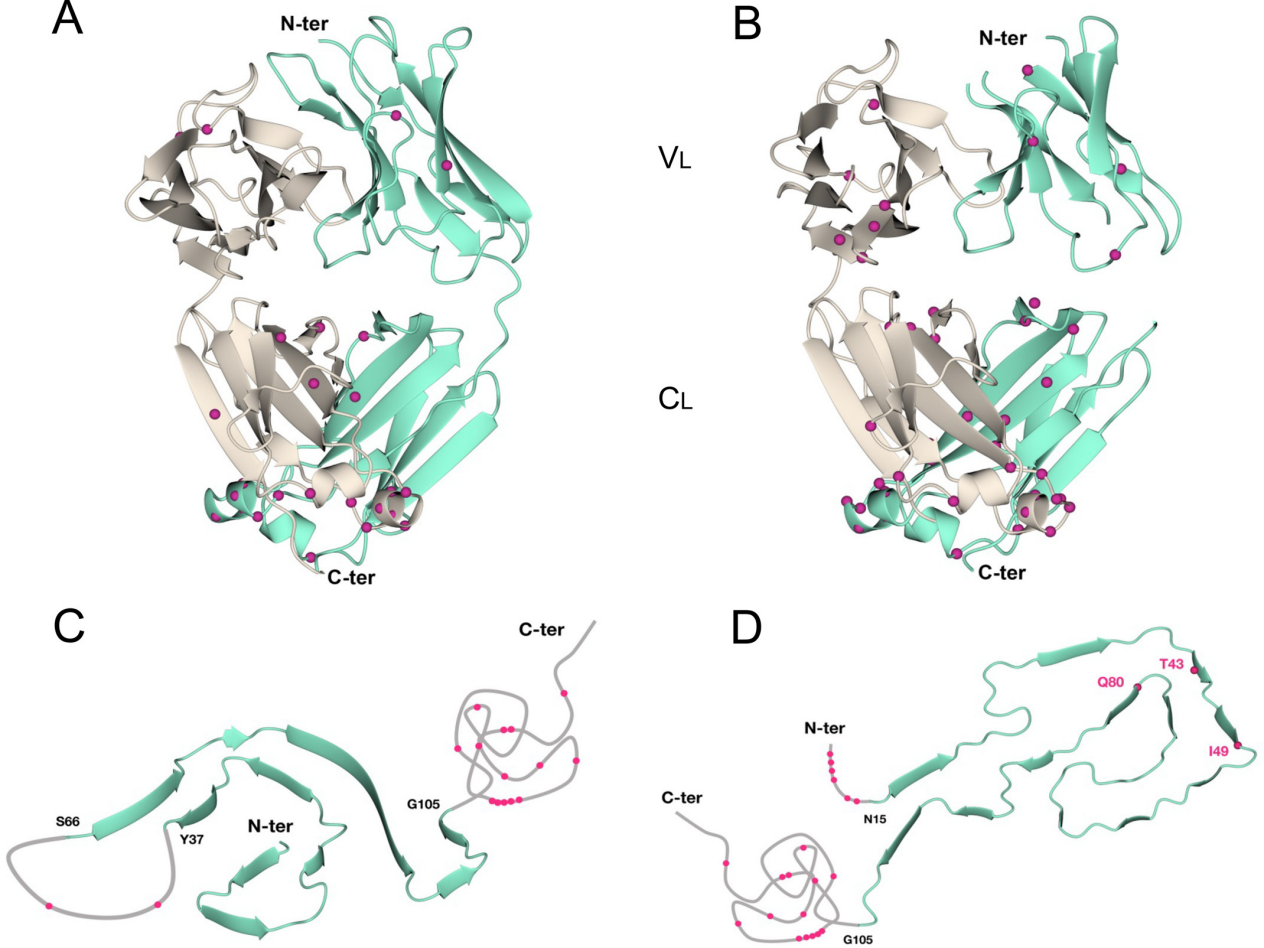
Mapping of the identified cleavage sites on the structures of (*A*) native JTO (PDB entry: 6MG4), (*B*) native AL-H7 (PDB entry: 5MUH), (*C*) fibrillar AL-55 (PDB entry: 6HUD), and (*D*) fibrillar λ1 LC (PDB entry: 6IC3).

To better understand the timing of the detected proteolytic events, the identified cleavage sites were mapped on the structures of native and fibrillar LCs. As indicated under “Experimental procedures,” the sites found in AL-55 were mapped on the native structure of JTO full-length dimer (24) and on the AL-55 fibrillar structure (17) (Fig. 5, *A* and *C*), whereas AL-H7 cleavage sites were mapped on the native structure of AL-H7 full-length dimer (26) and on the fibrillar structure of another λ1 LC belonging to the same *IGLV1* family VL (18) (Fig. 5, *B* and *D*).

In AL-55 amyloid deposits, only two proteolytic sites are located in the VL: in the native structure one is in an exposed loop and one is in a β strand, as shown in Table S1. On the other hand, the CL displays many more labeled sites, and they mainly map in the exposed α helix (residues 121–126) and in the buried dimer interface (residues 165–177). Intriguingly, none of the cleavage sites identified in this study maps in the AL-55 fibrillar core: the two VL sites lie in the 38–65 region, whereas all CL sites are located after residue 105 in the 106–214 region; both stretches are totally invisible, *i.e.* disordered, in the fibrillar structure.

On the other hand, in AL-H7 many more sites were found in the VL compared with AL-55. In the native structure, they are mostly located in the first 10 residues, which are either disordered or in β structure in the native AL-H7 dimer. Three further sites (in correspondence of residues 39, 45, and 76) are located in a partially exposed loop, in the hydrophobic core, and in an exposed loop, respectively. The positions of cleavage sites found in the CL of AL-H7 broadly resemble the ones found in AL-55 fibrils, such as the exposed α helix (residues 124–129) and the dimer interface or central β elements. It is noteworthy that, analogous to what was observed for AL-55 fibrillar structure, most of the AL-H7 proteolytic sites lie in regions outside the rigid structured fibrillar core of 6IC3: both the first fifteen-residue stretch and amino acids after 105 are located in regions invisible in the fibrillar core. The cleavages at position 39, 45, and 76 are the only exceptions to this pattern; in particular, residue 49 is nonsolvent exposed in the 6IC3 structure (Fig. 5*D*). Thus, these three cleavage sites are the only ones that cannot be explained from the analysis of the 6IC3 fibrillar structure. It should be noted, however, that the high-resolution structure of fibrillar AL-H7 is not available, and we can only assume that the structures of AL-H7 and of the 6IC3 λ1 fibrils are identical. In fact, it is also possible that Thr^39^, Ile^45^, and Gln^76^ are not buried in the actual structure of the AL-H7 fibril.

In summary, mapping on the native structures indicates that proteolytic sites lie on very diversified positions: exposed loop regions, β elements, and buried residues (Fig. 5, *A* and *B* and Table S1). Instead, with only three exceptions, all sites map on disordered and flexible regions away from the fibrillar core in both fibril structures (Fig. 5, *C* and *D* and Table S1).

We then analyzed the identified cleavage sites with the aim to detect possible patterns that could point toward the involvement of specific enzymes. Considering all identified cleavage sites, no definite predominance of particular residues was observed at the N-terminal side of the bonds, whereas serine was the most frequent identified residue at the C-terminal side (Fig. 4). Wide heterogeneity of the involved amino acids was otherwise observed (Fig. S4), preventing the identification of specific enzyme(s) that could explain such a complex proteolytic pattern. However, careful observation of the cleavage site distribution along the LCs' primary sequences shows that the pattern of digestion is heterogeneous: whereas sequence trimming is evident in some regions (N terminus of AL-H7 and beginning of the CL), cleavage sites are more dispersed in other regions. If the analysis is restricted to these scattered cleavage sites and to the extremities of the trimmed regions, marginal predominance of lysine residues at the N terminus of the cleaved bond was observed (Fig. 4).

## Discussion

Knowledge of the post-translational modifications affecting amyloid proteins *in vivo* is crucial to elucidate the mechanisms behind stability loss and fibril deposition. In addition to chemical modifications (*i.e.* oxidation (32, 33), deamidation (8, 34), and glycosylation (35)), fragmentation of the precursor protein is one of the most prominent aspects that characterize amyloid fibrils. On one hand, proteolysis has been implicated in amyloidogenesis in distinct forms of amyloidosis (10, 11, 12, 36, 37); on the other, post-deposition digestion may be involved in degradation of pathogenic aggregates (6, 22, 23).

In this study, we comprehensively characterized the ensemble of C- and N-terminal residues (N terminome and C terminome) of the LC fragments deposited as amyloid fibrils in the hearts of two patients affected by AL λ amyloidosis.

We homogenized samples in saline buffer to deplete tissue proteins and remove soluble prefibrillar LCs and contaminant Igs while retaining the insoluble amyloid fibrils (27). However, we did not proceed through the classical steps of amyloid extraction in water because of a number of considerations: in addition to the already mentioned low yield of fibrils from heart muscle in the absence of collagenase digestion (17, 18), high amounts of fibrils typically remain in the pellet even after extensive homogenization and water solubilization (27). Avoiding extensive purification procedures, therefore, ensures that all fibrils are included in the analysis. This approach implies a greater amount of carried-over tissue proteins, which is, however, manageable thanks to modern high-speed and high-resolution LC–MS/MS instrumentations and techniques.

This work adds novel significant information to the knowledge of the post-translational processing of amyloid LCs. More heterogeneous fragmentation points than were previously known were documented; the presence of the constant domain of the LCs in the fibrils was confirmed, firmly indicating that the full-length protein also participates in the aggregation process. Our data show that cleavage is more prominent in the CL compared with the VL. In fact, whereas fragmentation sites in VL are rare or, as in the case of AL-H7, concentrated in the N-terminal portion of the molecule, fragmentation in the CL is scattered along the sequence. The truncation points identified in the N-terminal portion of the CL (around positions 124–133) in these two samples are largely concordant with those previously reported in other studies (25). The more distal truncation points in the CL, however, are mostly novel and suggest an intense degradation activity on this portion of the molecule. Importantly, in the CL, the cleavage sites are largely overlapping between the two LCs (Fig. 4). Numerous labeled N termini were detected in this domain, representing the first amino acid of the corresponding peptide and suggesting the presence of relatively short fragments containing exclusively distal portions of the LC. This fact had been sporadically reported (21), but C-terminal fragments had gone unnoticed in the majority of studies, possibly in relation to their small size. Although the CL does not participate in the formation of the rigid and proteolysis-protected fibrillar core (17, 18), these CL fragments could be detected in our MS analyses; why they persist in the deposits and are not promptly degraded is, therefore, an open question. We cannot exclude that they encompass protein regions bound to other components, e.g. amyloid-associated proteins (28), which protect them from further degradation.

Some technical considerations should be drawn on our experimental approach, in light of which the results must be interpreted. First, the presence of some yet unidentified cleavage sites cannot be excluded. This can be attributed to factors including incomplete labeling (especially of carboxyl groups), possibly reduced efficiency of trypsin digestion in presence of dimethylated lysines, and need of caution in assigning C-terminal aspartate/glutamate residues and N-terminal lysines, unless double derivatization is observed. In addition, this study is not designed to provide quantitative information on the abundance and length of each fragment. Complementary studies based on top-down proteomic approaches will be important to further dissect the molecular weight and primary sequence of each fragment and to evaluate the abundance of each cleavage product relative to the other ones.

Overall, examination of the cleavage sites and mapping of the truncation points on the structures of native and fibrillar LCs allow drawing some considerations regarding the possible mechanisms and timing of proteolysis in relation to amyloid deposition. Taken together, our data do not allow identifying single specific proteases responsible for the global observed pattern. In fact, data are more consistent with the action of multiple endo- and exo-proteases acting simultaneously or sequentially to generate the proteolytic profile documented in the mature amyloid fibrils. The observed scenario could be generated by human proteases with broad substrate specificity, including cathepsins and matrix metalloproteases. In natively folded proteins, easily accessible proteolytic sites are typically exposed loops and, in general, loosely packed regions. Thus, had the proteolysis observed in AL-55 and AL-H7 occurred purely on the folded soluble dimers, most truncation points should have clustered in such regions; on the contrary, the proteolytic pattern observed in both AL-55 and AL-H7 mature fibrils is not compatible with such a model. Several proteolytic positions are located in buried regions or in β strands in the native structure, and such sites are nonideal for peptide bond hydrolysis. Previous work showed that amyloidogenic full-length LCs display specific biophysical traits compared with nonamyloidogenic ones: low fold and kinetic stability and high flexibility are typical of AL-associated LCs (16, 26, 38). Amyloidogenic LCs are more easily proteolyzed by model proteases compared with nonamyloidogenic ones (16, 24, 26, 38); however, in contrast with what was observed in this work, VL domains seem to be the most heavily proteolysed *in vitro* (38). Therefore, our data suggest to interpret such fast kinetics of proteolysis as an assessment of protein dynamics and not as a model of the molecular events preceding fibrillogenesis. It is also unlikely that proteolysis occurs on the unfolded state along the aggregation pathway, because this state is temporally very transient. Moreover, typically proteolysis of unfolded polypeptides is equally efficient along the whole sequence, resulting in formation of short peptides. Had proteolysis of the unfolded state had a crucial role in LC fibrillation, we would expect short, highly amyloidogenic peptides and not long stretches of the variable domain, as typically found in AL fibrils.

A totally different scenario can be found when the proteolytic sites are mapped on fibrillar structures. In AL-55 fibrils, all VL and CL proteolytic sites lie outside the rigid fibrillar core, suggestive of endoproteolytic events occurring after fibril deposition. Moreover, using the recently published structure of λ1 fibril (18) as a model for AL-H7 fibrils, the N-terminal “trimming” found in AL-H7 fibrils is compatible with the disordered N-terminal 15 residues in 6IC3 structure. Both in AL-55 and 6IC3 structures, CL domains are not part of the fibrillar core, indicating that they are unstructured or loosely structured. Accordingly, in the analyzed amyloid deposits, degradation of CL is significantly more extensive (this is consistent with the fact that the fragments visible by gel electrophoresis contain mostly VL). This consideration implies that the amyloid aggregates are the object of an intense degradation activity, which is however not effective in removing the accumulated fibrils.

Taken together, the observed fragmentation profile suggests that LC deposition as amyloid fibrils largely precedes the proteolytic events observed herein. However, our data do not rule out that proteolysis of native LC dimers in specific position(s) destabilizes their quaternary structure and triggers fibril formation, which would then involve both truncated and full-length LCs. Nevertheless, the precise length of this/these potential amyloidogenic fragment(s) may not be reliably identified from the analysis of natural AL amyloid material, which undergoes further extensive proteolytic remodeling on the poorly folded regions on the fibrillar surface. Nonetheless, in both heart samples analyzed here, few or no cleavage sites were identified on the variable or hinge regions; thus potential pre-fibrillogenesis cleavage events are rather likely to occur on the N-terminal portion of the constant domain.

The timing of fragmentation in AL has indeed been long debated, and distinct lines of evidence have suggested different conclusions. The presence of abundant LC fragments in the urines of AL patients (39) may suggest that these are circulating in the bloodstream and could be deposited into the fibrils. However, presence of LC fragments in AL patients' blood has never been consistently demonstrated, and MS evidence detected differences between urinary and deposited fragments (40). In addition, LC fragments with a MW similar to those found in tissues appear in urines after treatments that cause fibril disaggregation, such as 4′-iodo-4′-deoxydoxorubicin (41), suggesting that they may derive from reabsorbed deposits. Previous evidence also indicates that the fragmentation pattern differs across AL patients but is similar in distinct affected organs at the individual level (6); this may suggest that proteolysis precedes amyloidogenesis, because tissues differ in terms of protease expression. More recently, however, ultrastructural analysis of structural polymorphisms distribution has confirmed that fibrils in different organs are also similar at a structural level (42). This may translate into exposure of identical structural regions, which could be targeted by ubiquitous proteases. Defining the LC cleavage sites across tissues will provide further information to help assess whether the processing mechanisms are site-specific or ubiquitous. In light of what was previously reported for ATTR amyloidosis (43), it would also be important to investigate, on a wider population of patients, whether a correlation also exists between fragmentation pattern and disease phenotype in AL in terms of organ involvement and disease severity.

Complementing the present descriptive characterization with quantitative information on the abundance of the various fragments will be instrumental to provide further clues on the efficiency with which each bond is cleaved and, consequently, on the relative impact of specific enzymes in the processing mechanisms and on the timing and site where proteolysis occurs.

## Experimental procedures

### Chemicals

Solvents, HEPES, and MES (4-morpholineethanesulfonic acid) were purchased from VWR International; DTT and ethanolamine were purchased from Acros Organics (Geel, Belgium); PAA (MW 120,000–200,000) was purchased from Alfa Aesar; iodoacetamide (IAA), formaldehyde, PBS, sodium cyanoborohydride (NaBH_3_CN), *N*-hydroxysuccinimide (NHS), and EDC (*N*-ethyl-*N*′-(3-dimethylaminopropyl)carbodiimide hydrochloride) were purchased from Merck (Darmstadt, Germany); sequencing-grade modified trypsin (Trypsin Gold) was purchased from Promega; and cOmplete protease inhibitor mixture was purchased from Roche. All other chemicals were purchased from Merck.

### Enrichment of amyloid fibrils from diseased cardiac tissue

Myocardial tissue (left ventricle) was obtained from two patients (coded herein as AL-55 (17) and AL-H7 (26)) from the hearts removed, respectively, during autopsy examination and during cardiac transplantation. Both patients were affected by AL λ amyloidosis with severe cardiac involvement. After acquisition, tissues were placed at −80 °C without fixation and were stored frozen until use. Clinical data of both patients have been previously reported (17, 26). AL-55 and AL-H7 light chain sequences were derived from different germline genes, respectively *IGLV6-*57 and *IGLV1-*51. The sequences of the pathogenic light chains in the two patients are deposited in GenBank (AL-55: code MH670901 and AL-H7: code KC433671). The cryo-EM structure of *ex vivo* AL-55 fibril (PDB entry: 6HUD) and the crystallographic structure of soluble AL-H7 (PDB entry: 5MUH) are deposited in the Protein Data Bank.

All the procedures for fibril enrichment were performed on ice in presence of cOmplete protease inhibitor. Briefly, 0.5 g of tissue were diced in small pieces, washed 5 times in 1 ml of PBS, and then manually homogenized in Tris EDTA buffer (20 mm Tris, 140 mm NaCl, and 10 mm EDTA, pH 8.0) (17, 44) with a glass Potter pestle. The homogenate was centrifuged for 5 min at 3,100 × *g* at 4 °C and the pellet was retained. This step was repeated 10 times overall to remove the proteins soluble in the saline solution. The remaining pellet, containing abundant amyloid fibrils and residual tissue proteins, was retained for the proteomic analyses. Use of human samples for research purposes has been approved by the Ethical Committee of Fondazione IRCCS Policlinico San Matteo and was performed in accordance with the Declaration of Helsinki.

### One- and two-dimensional PAGE

For protein quantification, the pellet was incubated for 1 h with 8 m urea to dissolve protein aggregates. Protein samples were diluted and quantified using micro BCA assay (Thermo Fisher Scientific). The samples were analyzed by mono and bi-dimensional PAGE under denaturing and reducing conditions, as previously described (17). For immunoblotting, a rabbit polyclonal antiserum against human λ light chains (Agilent Dako, Santa Clara, CA, USA) was employed, as described (17).

### Protein sample processing and labeling of the free carboxyl and amino groups

200 µg of proteins were dissolved in 50 µl of 0.1 m HEPES, pH 7.5/4 m guanidinium-HCl (Gnd-HCl) for 1 h at room temperature (RT) and then diluted 1:2 with 0.1 m HEPES. Reduction of disulfide bonds (DTT final concentration 40 mm, 2 h, RT), alkylation of thiol groups (IAA final concentration 80 mm, 1 h, RT, dark), and quenching of residual IAA (adding fresh DTT to a concentration of 20 mm, 15 min, RT, dark) were performed before derivatizing primary amine groups by addition of formaldehyde (final concentration 40 mm) and NaBH_3_CN (final concentration 40 mm) (RT, overnight). This procedure resulted in dimethylation of primary amines; from now on, samples were split into two aliquots, one ready for the N-terminomics analysis and one to be destined to subsequent charge-reversal derivatization of free carboxyl groups. Both aliquots were then precipitated with ice-cold acetone (2 h, −20 °C), followed by one wash with acetone and one with methanol, and vacuum-dried. For the N-terminomics analysis, the protein pellets were reconstituted in 0.1 m HEPES/4 m Gnd-HCl (1 h, RT) and diluted with HEPES 0.1 m to obtain a final concentration of 0.5 m Gnd-HCl, followed by protein digestion with trypsin (1:20 w/w, 37 °C, ON), peptide purification on C18 ZipTips (Merck), vacuum drying, and resuspension in 10 µl of acetonitrile (ACN) 2% v/v/formic acid 0.1% v/v for LC–MS/MS.

For the C-terminomics labeling, C-terminal charge-reversal derivatization of the proteins previously subjected to amine protection (*i.e.* primary amine derivatization, previously described) was obtained by amidation of the free carboxyl groups with ethanolamine (45, 46). For this task, 100 µg of the previously precipitated samples were reconstituted in 100 µl of 0.1 m MES, pH 5/4 m Gnd-HCl (2 h, RT), and carboxyl groups were amidated by adding ethanolamine (final concentration 75 mm, previously buffered in 0.4 m MES, pH 5), EDC-HCl (freshly prepared; final concentration 50 mm), and NHS (freshly prepared; final concentration 10 mm). After overnight incubation at RT, the proteins were precipitated with acetone, washed, dissolved in 0.1 m HEPES, pH 7.5/4 m Gnd-HCl for 1 h, and then diluted 1:8 v/v with 0.1 m HEPES for digestion with trypsin (trypsin to protein ratio 1:20 w/w, 37 °C, ON). Digested samples were divided in two aliquots, one destined to PAA depletion of internal peptides, indicated as “PAA depletion,” and one to be analyzed without PAA depletion (“NO-PAA depletion”). NO-PAA depletion samples were stored at −80 °C, whereas in PAA depletion samples the trypsin-generated neo-N termini were protected by dimethylation (formaldehyde 20 mm and NaBH_3_CN 20 mm, 6 h, 37 °C). The peptides were then purified on SepPak C18 cartridges (Waters Corporation), vacuum-dried, dissolved in 100 µl of PAA 0.2 mm in MES 0.2 m, pH 5/Gnd-HCl 2 m/ACN 20% (previously filtered on 10-kDa filters), and sonicated for 30 s. Condensation between peptides and PAA was started by addition of EDC-HCl (final concentration 100 mm) and NHS (final concentration 20 mm). After overnight incubation at RT, the samples were filtered on 3-kDa filters to collect unbound peptides. Before LC–MS/MS analysis, all samples (both PAA depletion and NO-PAA depletion) were further purified on ZipTip C18, dried, and resuspended in 10 µl of ACN 2%/FA 0.1%.

### LC–MS/MS analysis and database search

LC–MS/MS analyses were performed on a Dionex UltiMate 3000 nano-UHPLC RSLC system coupled to a Q Exactive Plus mass spectrometer equipped with an EASY-spray ion source. Eight microliters of each sample were injected. The peptides were washed on a trap column (Acclaim Pepmap 100 C18, 0.3 × 5 mm, 5 µm, 100 Å) for 5 min with 2% ACN/0.1% FA at a flow rate of 10 μl/min and separated on an analytical column (PepMap RSLC C18, 75 µm × 50 cm, 2 µm, 100 Å) using a linear gradient (eluent A: 0.1% FA, eluent B: ACN/0.1% FA): 2–35% B in 61 min, 35–95% B in 12 min, and 95% B for 10 min, at a flow rate of 250 nl/min. Trap and column were maintained at 35 °C and the ion transfer capillary at 250 °C. Full mass spectra were acquired in positive ion mode over a 300–2000 *m*/*z* range and with a resolution setting of 70,000 full width at half maximum (at *m*/*z* 200). MS/MS events, acquired at a resolution of 17,500 full width at half maximum, were sequentially generated in a data-dependent manner on the top 10 most abundant precursor ions with charge ≥+2, selected with an isolation window of 2.0 *m*/*z*, fragmented by higher energy collisional dissociation with normalized collision energies of 30%, and dynamically excluded for 30 s.

Data were processed using the Sequest HT-based search engine contained in the Thermo Fisher Scientific Proteome Discoverer software, version 2.0. The following criteria were used for the identification of peptide sequences and related proteins: minimum precursor mass 350 Da; maximum precursor mass 5,000 Da (S/N ratio for peak filter 1.5); minimum peptide length six amino acids; maximum peptide length 144 amino acids; and tolerance on precursor mass 10 ppm and on fragment mass 0.03 Da. Percolator (maximum Δ Cn 0.05) was used for validation (based on q-value). Target false discovery rate was 0.01 in strict mode. Only peptides with high confidence and master proteins with ≥2 unique peptides were retained in results.

An iterative search strategy was employed. The first search was performed against the whole human protein database (74,190 entries, downloaded in August 2019 from Uniprot) with addition of the sequence of AL-H7 or AL-55 and with the following parameters: fully tryptic enzymatic cleavage, two maximum missed cleavages, carbamidomethylation of cysteines, and dimethylation of peptide N termini as static modifications. Proteins identified in this step were selected for creation of a “target database” used in the second search, in which enzymatic cleavage was changed to semitryptic and modifications were increased to four maximum equal modifications per peptide and five maximum dynamic modifications per peptide. Carbamidomethylation of cysteines was also set as static modification in these second search runs. A few additional parameters instead differed between the second search used in N-terminomic and C-terminomic identification. For the N-terminomics study, dimethylation of lysines and peptide N termini (Δ mass +28.031), acetylation on peptide N termini, and cyclization of N-terminal glutamine were set as variable modifications. It is of note that, for peptides with a lysine residue at the N terminus, it is not possible to distinguish whether a derivatization occurs on the N-terminal or on the ε-amino group unless both sites are modified. For the C-terminomic study, dimethylation of lysines and peptide N termini and amidation with ethanolamine (Δ mass +43.042) on aspartate and glutamate and on peptide C termini were set as dynamic modifications. All spectra from the derivatized peptides were manually confirmed. Because aspartate and glutamate residues are also labeled in the side chain carboxyl group, peptides ending with aspartate or glutamate were considered derivatized at the C terminus only if modification occurred both on the C terminus and on the side chain.

### Mapping of the cleavage sites on the available LC structures

The cleavage sites identified on AL-55 were mapped on the already available native structure of JTO full-length LC dimer (PDB entry: 6MG4) (24), which, as AL-55, belongs to the λ6 isotype and displays 90% sequence identity with AL-55, and on the AL-55 fibrillar structure recently reported (PDB entry: 6HUD) (17) (Fig. 5, *A* and *C*). AL-H7 cleavage sites were instead mapped on the native structure of AL-H7 full-length dimer (PDB entry: 5MUH) (26) and on the fibrillar structure of another λ1 LC belonging to the same *IGLV1* family VL and with a sequence identity of 70% (PDB entry: 6IC3) (18) with AL-H7 (Fig. 5, *B* and *D*). Possible enzymes responsible for the cleavages were searched using the MEROPS bioinformatics resource (RRID:SCR_007777) (47).

## Data availability

The mass spectrometry proteomics data have been deposited to the Proteome Xchange Consortium via the PRIDE partner repository (48) with the dataset identifier PXD020858. Other raw data will be shared upon request (contact Francesca Lavatelli, E-mail: francesca.lavatelli@unipv.it).

10.13039/501100002803Fondazione Cariplo (Cariplo Foundation) (2015-0591) to Francesca Lavatelli, Stefano Ricagno, and Giovanni Palladini
10.13039/501100002803Fondazione Cariplo (Cariplo Foundation) (2016-0489) to Francesca Lavatelli, Stefano Ricagno, and Giovanni Palladini
10.13039/501100005010Associazione Italiana per la Ricerca sul Cancro (AIRC) (9965) to Giampaolo Merlini
10.13039/501100003196Ministero della Salute (Ministry of Health, Italy) (RF-2013-02355259) to Giampaolo Merlini, and Giovanni Palladini
10.13039/501100003196Ministero della Salute (Ministry of Health, Italy) (RF-2016-02361756) to Giampaolo Merlini, and Giovanni Palladini
10.13039/501100003197Ministry of Health, Italy | Agenzia Italiana del Farmaco, Ministero della Salute (AIFA) (AIFA-2016-02364602) to Giovanni Palladini
EUROPEAN JOINT PROGRAMME ON RARE DISEASES (E-RARE JTC 2016 grant ReDox) to Giovanni Palladini
