# Supplementary material for: Mass spectrometry characterization of light chain fragmentation sites in cardiac AL amyloidosis: insights into the timing of proteolysis
Source: J Biol Chem. 2021 Jan 13;295(49):16572–84. doi: 10.1074/jbc.RA120.013461 (PMC7864057; doi:10.1074/jbc.RA120.013461)
Supplement: Supplementary file 1 [file mmc1.pdf]

Supporting Information

Figure S1. N- and C-terminal labeling of proteins other than light chains identified in the LC-MS/MS analysis: (A) nearly 100% of all the detected proteins in both samples presented a labeled N-terminus, either canonical or non canonical (fragmented); (B) approximately half of all the detected proteins in both samples presented a labeled C-terminus, either canonical or fragmented.

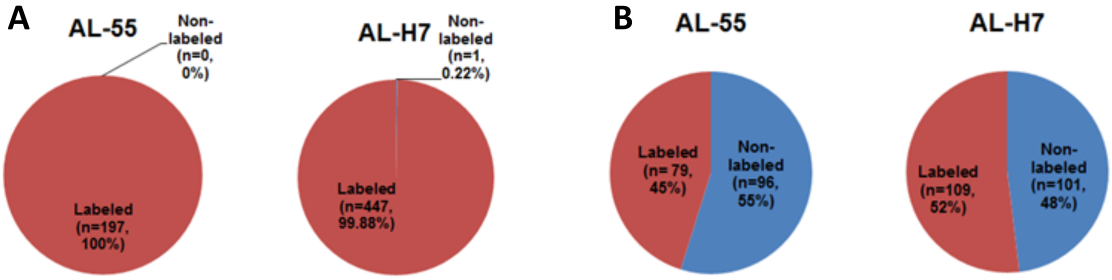

Figure S2. Annotated spectra of all the peptides containing labeled N-termini of AL-55 (A) and AL-H7 (B) and C-termini of AL-55 (C) and AL-H7 (D).

A

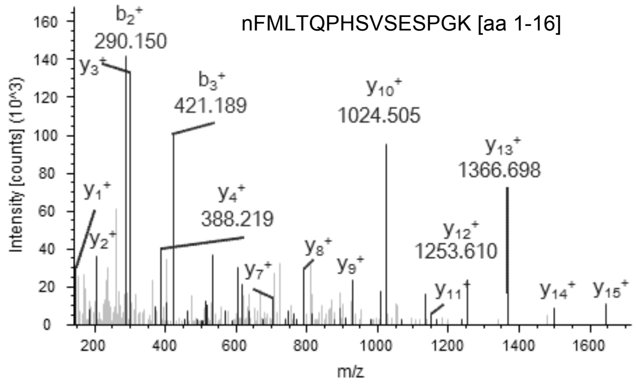

#1	Immonium	b⁺	Seq.	y⁺	#2
1	115.087	143.082	N-Dimethyl		16
2	120.081	290.150	F	1644.805	15
3	104.053	421.190	M	1497.737	14
4	86.096	534.274	L	1366.696	13
5	74.060	635.322	T	1253.612	12
6	101.071	763.381	Q	1152.564	11
7	70.065	860.433	P	1024.506	10
8	110.071	997.492	H	927.453	9
9	60.044	1084.524	S	790.394	8
10	72.081	1183.593	V	703.362	7
11	60.044	1270.625	S	604.294	6
12	102.055	1399.667	E	517.262	5
13	60.044	1486.699	S	388.219	4
14	70.065	1583.752	P	301.187	3
15	30.034	1640.774	G	204.134	2
16	101.107		K	147.113	1

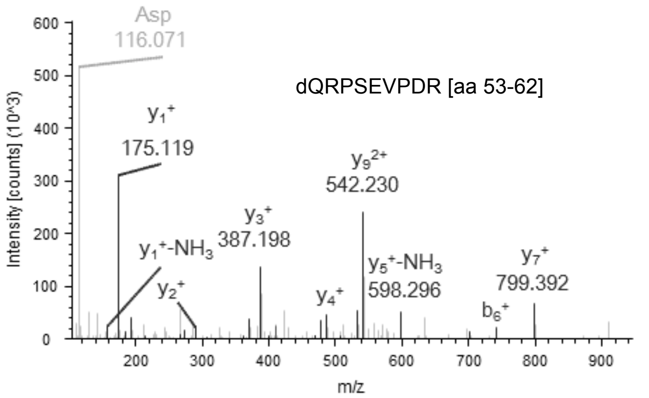

#1	Immonium	b⁺	Seq.	y⁺	#2
1	116.071	144.066	D-Dimethyl		10
2	101.071	272.124	Q	1083.554	9
3	129.113	428.225	R	955.496	8
4	70.065	525.278	P	799.394	7
5	60.044	612.310	S	702.342	6
6	102.055	741.353	E	615.310	5
7	72.081	840.421	V	486.267	4
8	70.065	937.474	P	387.199	3
9	88.039	1052.501	D	290.146	2
10	129.113		R	175.119	1

Figure S2 continued

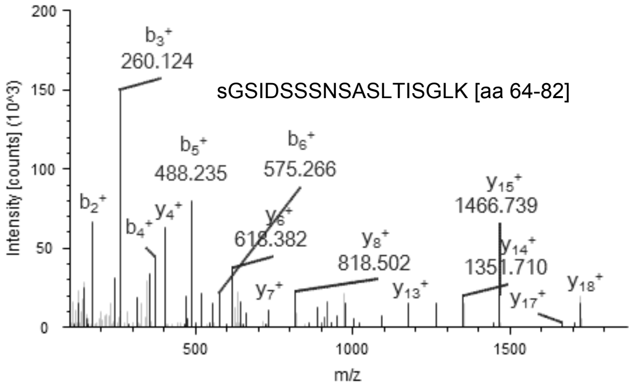

#1	Immonium	b⁺	Seq.	y⁺	#2
1	88.076	116.071	S-Dimethyl		19
2	30.034	173.092	G	1723.871	18
3	60.044	260.124	S	1666.849	17
4	86.096	373.208	I	1579.817	16
5	88.039	488.235	D	1466.733	15
6	60.044	575.267	S	1351.706	14
7	60.044	662.299	S	1264.674	13
8	60.044	749.331	S	1177.642	12
9	87.055	863.374	N	1090.610	11
10	60.044	950.406	S	976.567	10
11	44.049	1021.443	A	889.535	9
12	60.044	1108.475	S	818.498	8
13	86.096	1221.559	L	731.466	7
14	74.060	1322.607	T	618.382	6
15	86.096	1435.691	I	517.334	5
16	60.044	1522.723	S	404.250	4
17	30.034	1579.745	G	317.218	3
18	86.096	1692.829	L	260.197	2
19	101.107		K	147.113	1

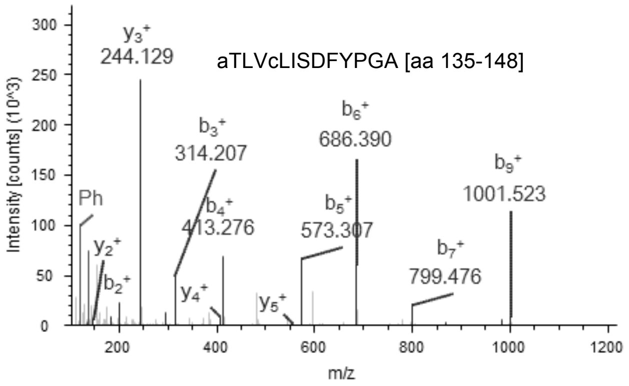

#1	Immonium	b⁺	Seq.	y⁺	#2
1	72.081	100.076	A-Dimethyl		14
2	74.060	201.123	T	1455.719	13
3	86.096	314.207	L	1354.671	12
4	72.081	413.276	V	1241.587	11
5	133.043	573.307	C-Carbamidomethyl	1142.519	10
6	86.096	686.391	L	982.488	9
7	86.096	799.475	I	869.404	8
8	60.044	886.507	S	756.320	7
9	88.039	1001.534	D	669.288	6
10	120.081	1148.602	F	554.261	5
11	136.076	1311.665	Y	407.193	4
12	70.065	1408.718	P	244.129	3
13	30.034	1465.740	G	147.076	2
14	44.049		A	90.055	1

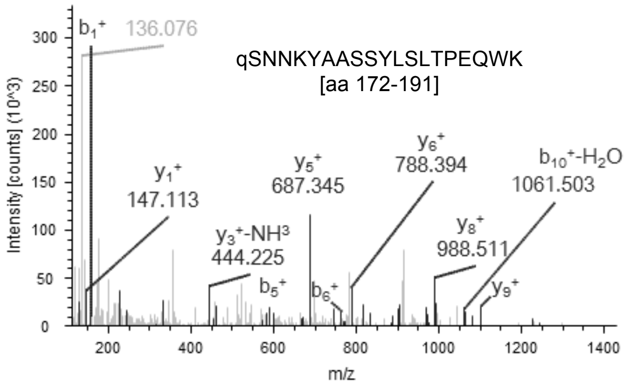

#1	Immonium	b⁺	Seq.	y⁺	#2
1	129.102	157.097	Q-Dimethyl		20
2	60.044	244.129	S	2187.072	19
3	87.055	358.172	N	2100.040	18
4	87.055	472.215	N	1985.997	17
5	101.107	600.310	K	1871.954	16
6	136.076	763.373	Y	1743.859	15
7	44.049	834.410	A	1580.796	14
8	44.049	905.448	A	1509.758	13
9	60.044	992.480	S	1438.721	12
10	60.044	1079.512	S	1351.689	11
11	136.076	1242.575	Y	1264.657	10
12	86.096	1355.659	L	1101.594	9
13	60.044	1442.691	S	988.510	8
14	86.096	1555.775	L	901.478	7
15	74.060	1656.823	T	788.394	6
16	70.065	1753.876	P	687.346	5
17	102.055	1882.918	E	590.293	4
18	101.071	2010.977	Q	461.251	3
19	159.092	2197.056	W	333.192	2
20	101.107		K	147.113	1

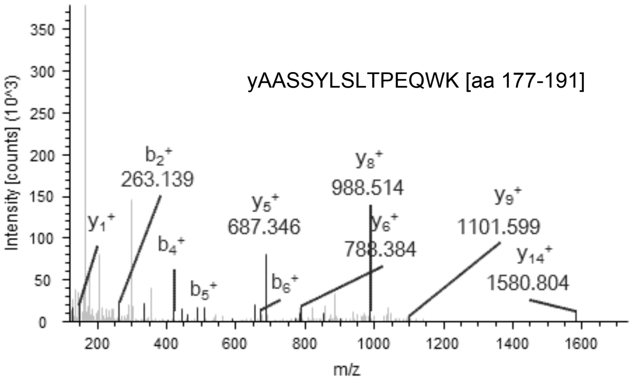

#1	Immonium	b⁺	Seq.	y⁺	#2
1	164.107	192.102	Y-Dimethyl		15
2	44.049	263.139	A	1580.796	14
3	44.049	334.176	A	1509.758	13
4	60.044	421.208	S	1438.721	12
5	60.044	508.240	S	1351.689	11
6	136.076	671.304	Y	1264.657	10
7	86.096	784.388	L	1101.594	9
8	60.044	871.420	S	988.510	8
9	86.096	984.504	L	901.478	7
10	74.060	1085.551	T	788.394	6
11	70.065	1182.604	P	687.346	5
12	102.055	1311.647	E	590.293	4
13	101.071	1439.705	Q	461.251	3
14	159.092	1625.785	W	333.192	2
15	101.107		K	147.113	1

Figure S2 continued

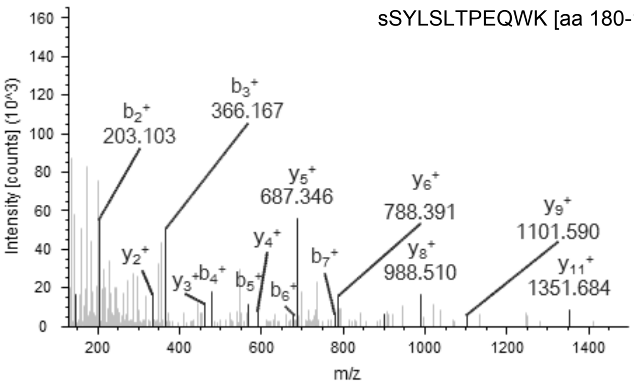

#1	Immonium	b+	Seq.	y+	#2
1	88.076	116.071	S-Dimethyl		12
2	60.044	203.103	S	1351.689	11
3	136.076	366.166	Y	1264.657	10
4	86.096	479.250	L	1101.594	9
5	60.044	566.282	S	988.510	8
6	86.096	679.366	L	901.478	7
7	74.060	780.414	T	788.394	6
8	70.065	877.467	P	687.346	5
9	102.055	1006.509	E	590.293	4
10	101.071	1134.568	Q	461.251	3
11	159.092	1320.647	W	333.192	2
12	101.107		K	147.113	1

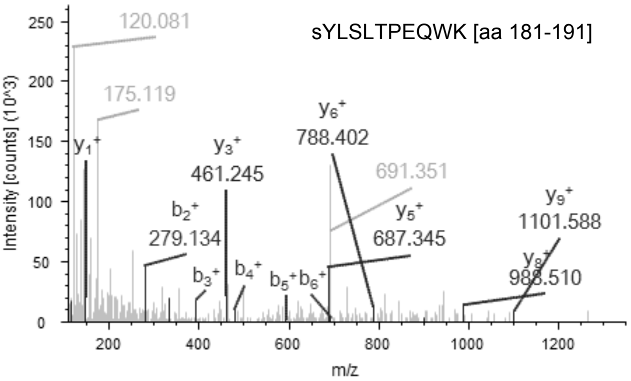

#1	Immonium	b+	Seq.	y+	#2
1	88.076	116.071	S-Dimethyl		11
2	136.076	279.134	Y	1264.657	10
3	86.096	392.218	L	1101.594	9
4	60.044	479.250	S	988.510	8
5	86.096	592.334	L	901.478	7
6	74.060	693.382	T	788.394	6
7	70.065	790.435	P	687.346	5
8	102.055	919.477	E	590.293	4
9	101.071	1047.536	Q	461.251	3
10	159.092	1233.615	W	333.192	2
11	101.107		K	147.113	1

B

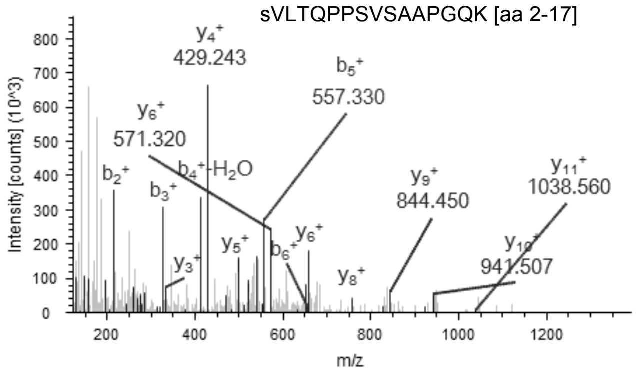

#1	Immonium	b+	Seq.	y+	#2
1	88.076	116.071	S-Dimethyl		16
2	72.081	215.139	V	1479.817	15
3	86.096	328.223	L	1380.748	14
4	74.060	429.271	T	1267.664	13
5	101.071	557.329	Q	1166.616	12
6	70.065	654.382	P	1038.558	11
7	70.065	751.435	P	941.505	10
8	60.044	838.467	S	844.452	9
9	72.081	937.535	V	757.420	8
10	60.044	1024.567	S	658.352	7
11	44.049	1095.604	A	571.320	6
12	44.049	1166.642	A	500.283	5
13	70.065	1263.694	P	429.246	4
14	30.034	1320.716	G	332.193	3
15	101.071	1448.774	Q	275.171	2
16	101.107		K	147.113	1

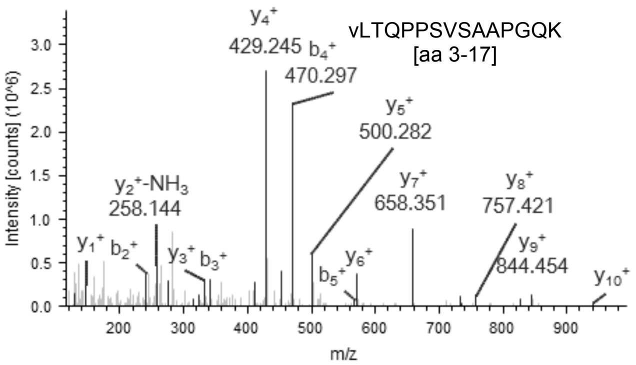

#1	Immonium	b+	Seq.	y+	#2
1	100.112	128.107	V-Dimethyl		15
2	86.096	241.191	L	1380.748	14
3	74.060	342.239	T	1267.664	13
4	101.071	470.297	Q	1166.616	12
5	70.065	567.350	P	1038.558	11
6	70.065	664.403	P	941.505	10
7	60.044	751.435	S	844.452	9
8	72.081	850.503	V	757.420	8
9	60.044	937.535	S	658.352	7
10	44.049	1008.572	A	571.320	6
11	44.049	1079.610	A	500.283	5
12	70.065	1176.662	P	429.246	4
13	30.034	1233.684	G	332.193	3
14	101.071	1361.742	Q	275.171	2
15	101.107		K	147.113	1

Figure S2 continued

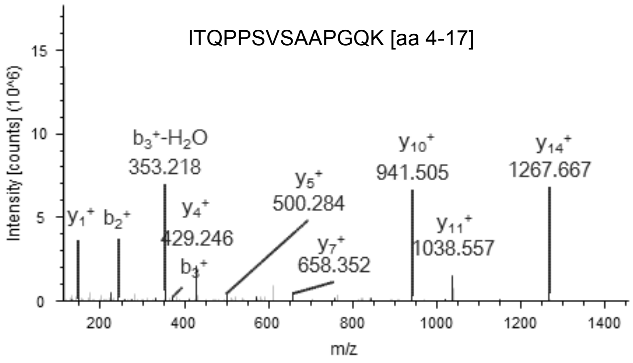

#1	Immonium	b⁺	Seq.	y⁺	#2
1	114.128	142.123	L-Dimethyl		14
2	74.060	243.170	T	1267.664	13
3	101.071	371.229	Q	1166.616	12
4	70.065	468.282	P	1038.558	11
5	70.065	565.334	P	941.505	10
6	60.044	652.366	S	844.452	9
7	72.081	751.435	V	757.420	8
8	60.044	838.467	S	658.352	7
9	44.049	909.504	A	571.320	6
10	44.049	980.541	A	500.283	5
11	70.065	1077.594	P	429.246	4
12	30.034	1134.615	G	332.193	3
13	101.071	1262.674	Q	275.171	2
14	101.107		K	147.113	1

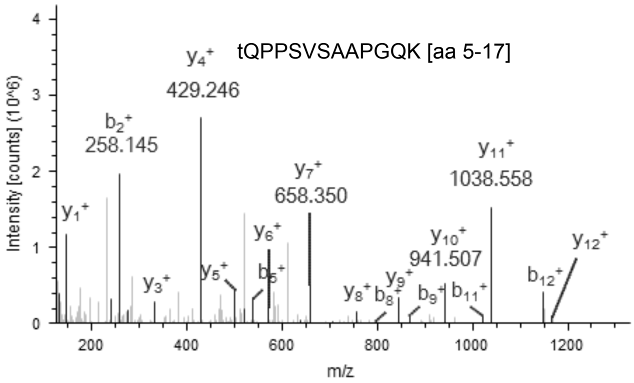

#1	Immonium	b⁺	Seq.	y⁺	#2
1	102.091	130.086	T-Dimethyl		13
2	101.071	258.145	Q	1166.616	12
3	70.065	355.198	P	1038.558	11
4	70.065	452.250	P	941.505	10
5	60.044	539.282	S	844.452	9
6	72.081	638.351	V	757.420	8
7	60.044	725.383	S	658.352	7
8	44.049	796.420	A	571.320	6
9	44.049	867.457	A	500.283	5
10	70.065	964.510	P	429.246	4
11	30.034	1021.531	G	332.193	3
12	101.071	1149.590	Q	275.171	2
13	101.107		K	147.113	1

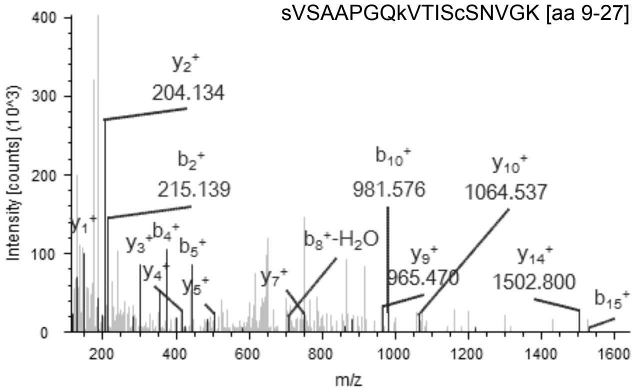

#1	Immonium	b⁺	Seq.	y⁺	#2
1	88.076	116.071	S-Dimethyl		19
2	72.081	215.139	V	1830.974	18
3	60.044	302.171	S	1731.906	17
4	44.049	373.208	A	1644.874	16
5	44.049	444.245	A	1573.837	15
6	70.065	541.298	P	1502.800	14
7	30.034	598.320	G	1405.747	13
8	101.071	726.378	Q	1348.725	12
9	129.139	882.504	K-Dimethyl	1220.667	11
10	72.081	981.573	V	1064.540	10
11	74.060	1082.620	T	965.472	9
12	86.096	1195.705	I	864.424	8
13	60.044	1282.737	S	751.340	7
14	133.043	1442.767	C-Carbamidomethyl	664.308	6
15	60.044	1529.799	S	504.278	5
16	87.055	1643.842	N	417.246	4
17	72.081	1742.911	V	303.203	3
18	30.034	1799.932	G	204.134	2
19	101.107		K	147.113	1

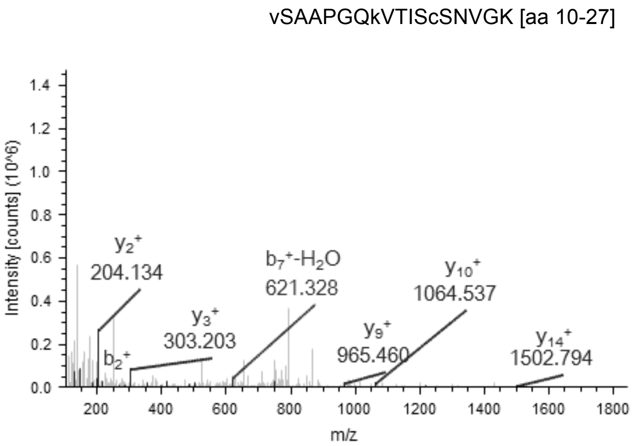

#1	Immonium	b⁺	Seq.	y⁺	#2
1	100.112	128.107	V-Dimethyl		18
2	60.044	215.139	S	1731.906	17
3	44.049	286.176	A	1644.874	16
4	44.049	357.213	A	1573.837	15
5	70.065	454.266	P	1502.800	14
6	30.034	511.287	G	1405.747	13
7	101.071	639.346	Q	1348.725	12
8	129.139	795.472	K-Dimethyl	1220.667	11
9	72.081	894.541	V	1064.540	10
10	74.060	995.588	T	965.472	9
11	86.096	1108.672	I	864.424	8
12	60.044	1195.705	S	751.340	7
13	133.043	1355.735	C-Carbamidomethyl	664.308	6
14	60.044	1442.767	S	504.278	5
15	87.055	1556.810	N	417.246	4
16	72.081	1655.879	V	303.203	3
17	30.034	1712.900	G	204.134	2
18	101.107		K	147.113	1

Figure S2 continued

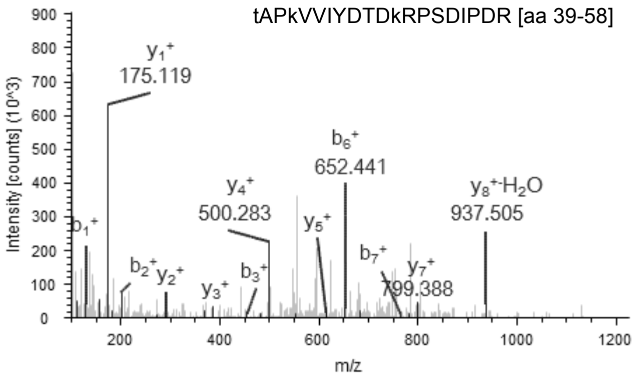

#1	Immonium	b+	Seq.	y+	#2
1	102.091	130.086	T-Dimethyl		20
2	44.049	201.123	A	2241.224	19
3	70.065	298.176	P	2170.187	18
4	129.139	454.302	K-Dimethyl	2073.134	17
5	72.081	553.371	V	1917.008	16
6	72.081	652.439	V	1817.939	15
7	86.096	765.523	I	1718.871	14
8	136.076	928.587	Y	1605.787	13
9	88.039	1043.614	D	1442.723	12
10	74.060	1144.661	T	1327.696	11
11	88.039	1259.688	D	1226.649	10
12	129.139	1415.814	K-Dimethyl	1111.622	9
13	129.113	1571.916	R	955.496	8
14	70.065	1668.968	P	799.394	7
15	60.044	1756.000	S	702.342	6
16	88.039	1871.027	D	615.310	5
17	86.096	1984.111	I	500.283	4
18	70.065	2081.164	P	387.199	3
19	88.039	2196.191	D	290.146	2
20	129.113		R	175.119	1

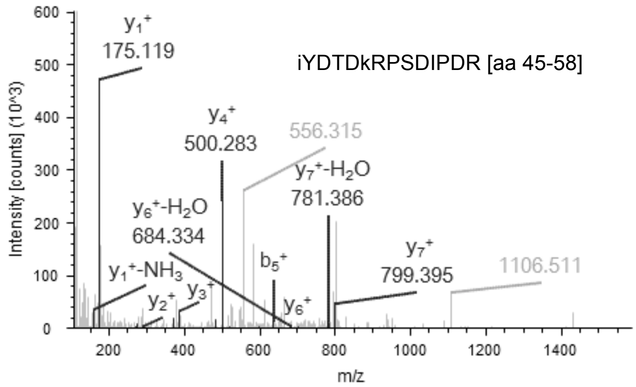

#1	Immonium	b+	Seq.	y+	#2
1	114.128	142.123	I-Dimethyl		14
2	136.076	305.186	Y	1605.787	13
3	88.039	420.213	D	1442.723	12
4	74.060	521.261	T	1327.696	11
5	88.039	636.288	D	1226.649	10
6	129.139	792.414	K-Dimethyl	1111.622	9
7	129.113	948.515	R	955.496	8
8	70.065	1045.568	P	799.394	7
9	60.044	1132.600	S	702.342	6
10	88.039	1247.627	D	615.310	5
11	86.096	1360.711	I	500.283	4
12	70.065	1457.763	P	387.199	3
13	88.039	1572.790	D	290.146	2
14	129.113		R	175.119	1

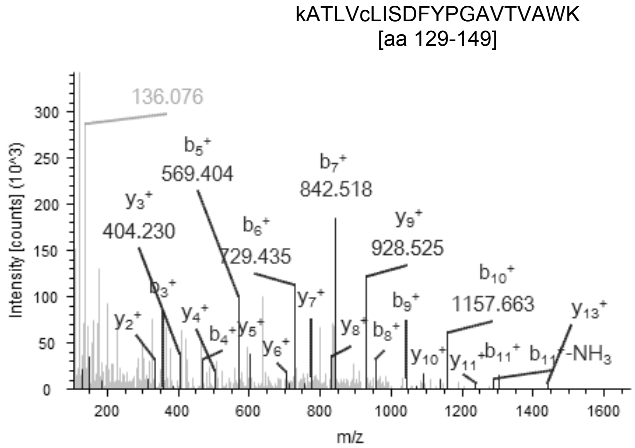

#1	Immonium	b+	Seq.	y+	#2
1	157.170	185.165	K-Dimethyl-Dimethyl		21
2	44.049	256.202	A	2211.152	20
3	74.060	357.250	T	2140.115	19
4	86.096	470.334	L	2039.067	18
5	72.081	569.402	V	1925.983	17
6	133.043	729.433	C-Carbamidomethyl	1826.915	16
7	86.096	842.517	L	1666.884	15
8	86.096	955.601	I	1553.800	14
9	60.044	1042.633	S	1440.716	13
10	88.039	1157.660	D	1353.684	12
11	120.081	1304.728	F	1238.657	11
12	136.076	1467.792	Y	1091.588	10
13	70.065	1564.844	P	928.525	9
14	30.034	1621.866	G	831.472	8
15	44.049	1692.903	A	774.451	7
16	72.081	1791.971	V	703.414	6
17	74.060	1893.019	T	604.345	5
18	72.081	1992.087	V	503.298	4
19	44.049	2063.125	A	404.229	3
20	159.092	2249.204	W	333.192	2
21	101.107		K	147.113	1

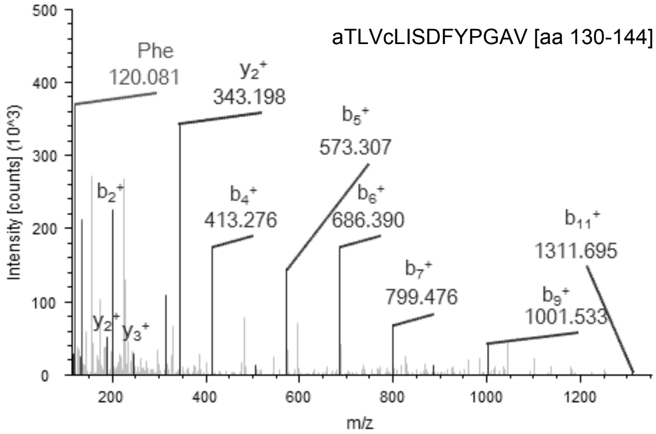

#1	Immonium	b+	Seq.	y+	#2
1	72.081	100.076	A-Dimethyl		15
2	74.060	201.123	T	1554.787	14
3	86.096	314.207	L	1453.740	13
4	72.081	413.276	V	1340.656	12
5	133.043	573.307	C-Carbamidomethyl	1241.587	11
6	86.096	686.391	L	1081.556	10
7	86.096	799.475	I	968.472	9
8	60.044	886.507	S	855.388	8
9	88.039	1001.534	D	768.356	7
10	120.081	1148.602	F	653.329	6
11	136.076	1311.665	Y	506.261	5
12	70.065	1408.718	P	343.198	4
13	30.034	1465.740	G	246.145	3
14	44.049	1536.777	A	189.123	2
15	72.081		V	118.086	1

Figure S2 continued

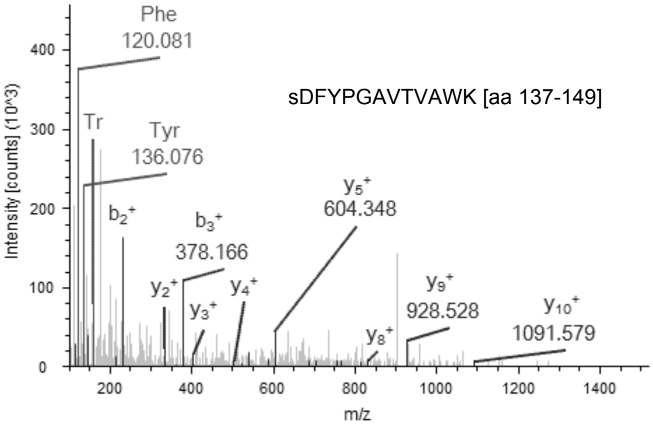

#1	Immonium	b+	Seq.	y+	#2
1	88.076	116.071	S-Dimethyl		13
2	88.039	231.098	D	1353.684	12
3	120.081	378.166	F	1238.657	11
4	136.076	541.229	Y	1091.588	10
5	70.065	638.282	P	928.525	9
6	30.034	695.304	G	831.472	8
7	44.049	766.341	A	774.451	7
8	72.081	865.409	V	703.414	6
9	74.060	966.457	T	604.345	5
10	72.081	1065.525	V	503.298	4
11	44.049	1136.562	A	404.229	3
12	159.092	1322.642	W	333.192	2
13	101.107		K	147.113	1

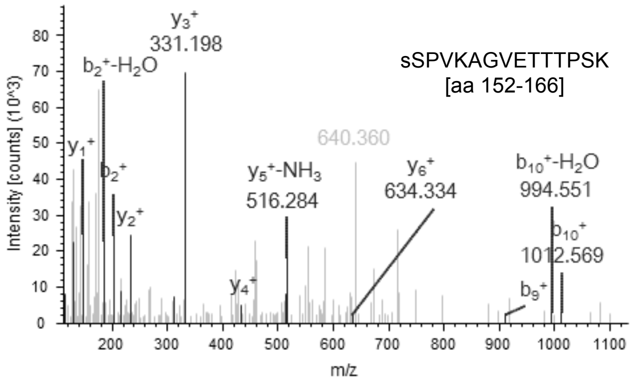

#1	Immonium	b+	Seq.	y+	#2
1	88.076	116.071	S-Dimethyl		15
2	60.044	203.103	S	1429.790	14
3	70.065	300.155	P	1342.758	13
4	72.081	399.224	V	1245.705	12
5	129.139	555.350	K-Dimethyl	1146.636	11
6	44.049	626.387	A	990.510	10
7	30.034	683.409	G	919.473	9
8	72.081	782.477	V	862.452	8
9	102.055	911.520	E	763.383	7
10	74.060	1012.567	T	634.341	6
11	74.060	1113.615	T	533.293	5
12	74.060	1214.663	T	432.245	4
13	70.065	1311.715	P	331.198	3
14	60.044	1398.747	S	234.145	2
15	101.107		K	147.113	1

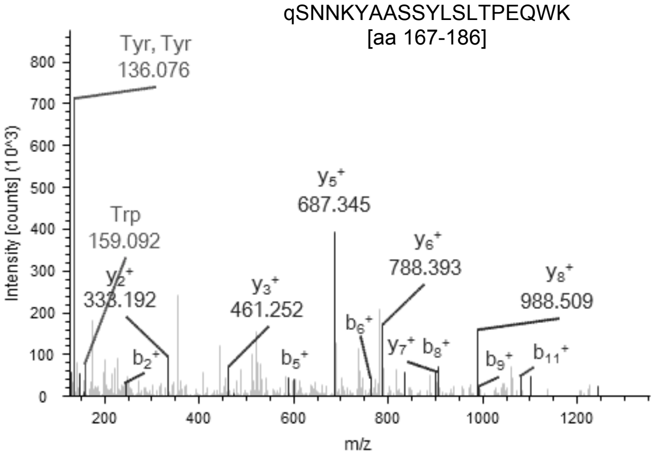

#1	Immonium	b+	Seq.	y+	#2
1	129.102	157.097	Q-Dimethyl		20
2	60.044	244.129	S	2187.072	19
3	87.055	358.172	N	2100.040	18
4	87.055	472.215	N	1985.997	17
5	101.107	600.310	K	1871.954	16
6	136.076	763.373	Y	1743.859	15
7	44.049	834.410	A	1580.796	14
8	44.049	905.448	A	1509.758	13
9	60.044	992.480	S	1438.721	12
10	60.044	1079.512	S	1351.689	11
11	136.076	1242.575	Y	1264.657	10
12	86.096	1355.659	L	1101.594	9
13	60.044	1442.691	S	988.510	8
14	86.096	1555.775	L	901.478	7
15	74.060	1656.823	T	788.394	6
16	70.065	1753.876	P	687.346	5
17	102.055	1882.918	E	590.293	4
18	101.071	2010.977	Q	461.251	3
19	159.092	2197.056	W	333.192	2
20	101.107		K	147.113	1

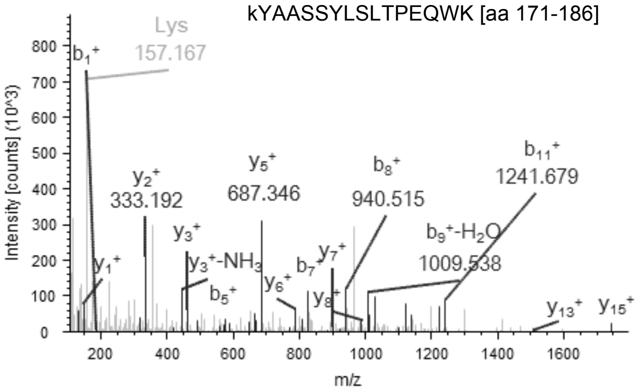

#1	Immonium	b+	Seq.	y+	#2
1	157.170	185.165	K-Dimethyl-Dimethyl		16
2	136.076	348.228	Y	1743.859	15
3	44.049	419.265	A	1580.796	14
4	44.049	490.302	A	1509.758	13
5	60.044	577.334	S	1438.721	12
6	60.044	664.366	S	1351.689	11
7	136.076	827.430	Y	1264.657	10
8	86.096	940.514	L	1101.594	9
9	60.044	1027.546	S	988.510	8
10	86.096	1140.630	L	901.478	7
11	74.060	1241.678	T	788.394	6
12	70.065	1338.730	P	687.346	5
13	102.055	1467.773	E	590.293	4
14	101.071	1595.832	Q	461.251	3
15	159.092	1781.911	W	333.192	2
16	101.107		K	147.113	1

Figure S2 continued

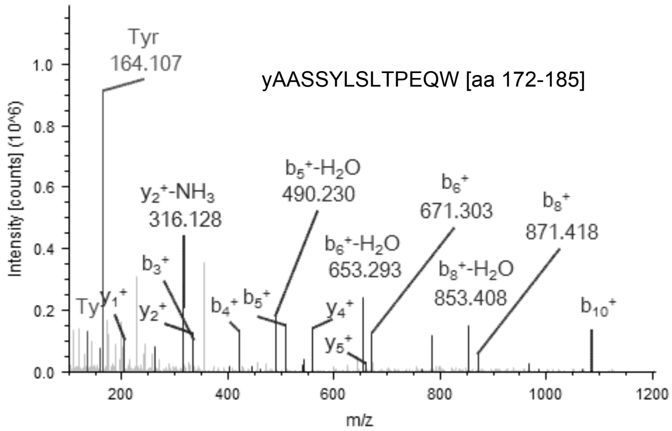

#1	Immonium	b⁺	Seq.	y⁺	#2
1	164.107	192.102	Y-Dimethyl		14
2	44.049	263.139	A	1452.701	13
3	44.049	334.176	A	1381.663	12
4	60.044	421.208	S	1310.626	11
5	60.044	508.240	S	1223.594	10
6	136.076	671.304	Y	1136.562	9
7	86.096	784.388	L	973.499	8
8	60.044	871.420	S	860.415	7
9	86.096	984.504	L	773.383	6
10	74.060	1085.551	T	660.299	5
11	70.065	1182.604	P	559.251	4
12	102.055	1311.647	E	462.198	3
13	101.071	1439.705	Q	333.156	2
14	159.092		W	205.097	1

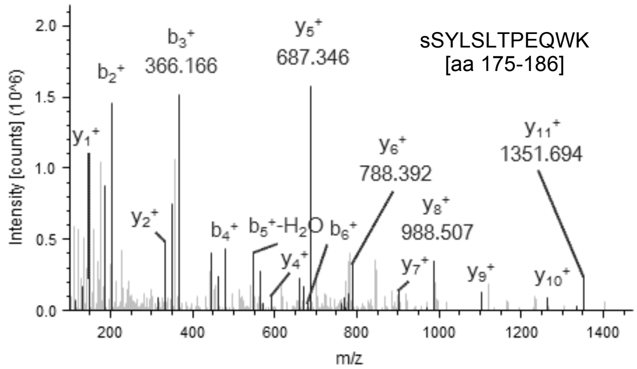

#1	Immonium	b⁺	Seq.	y⁺	#2
1	88.076	116.071	S-Dimethyl		12
2	60.044	203.103	S	1351.689	11
3	136.076	366.166	Y	1264.657	10
4	86.096	479.250	L	1101.594	9
5	60.044	566.282	S	988.510	8
6	86.096	679.366	L	901.478	7
7	74.060	780.414	T	788.394	6
8	70.065	877.467	P	687.346	5
9	102.055	1006.509	E	590.293	4
10	101.071	1134.568	Q	461.251	3
11	159.092	1320.647	W	333.192	2
12	101.107		K	147.113	1

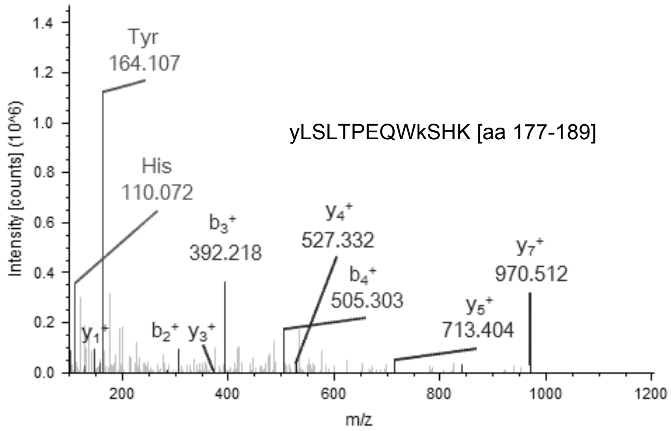

#1	Immonium	b⁺	Seq.	y⁺	#2
1	164.107	192.102	Y-Dimethyl		13
2	86.096	305.186	L	1481.811	12
3	60.044	392.218	S	1368.727	11
4	86.096	505.302	L	1281.695	10
5	74.060	606.350	T	1168.611	9
6	70.065	703.403	P	1067.563	8
7	102.055	832.445	E	970.510	7
8	101.071	960.504	Q	841.468	6
9	159.092	1146.583	W	713.409	5
10	129.139	1302.709	K-Dimethyl	527.330	4
11	60.044	1389.741	S	371.204	3
12	110.071	1526.800	H	284.172	2
13	101.107		K	147.113	1

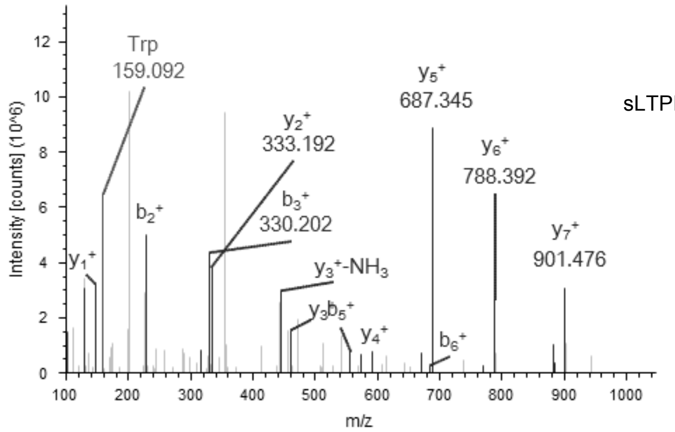

#1	Immonium	b⁺	Seq.	y⁺	#2
1	88.076	116.071	S-Dimethyl		8
2	86.096	229.155	L	901.478	7
3	74.060	330.202	T	788.394	6
4	70.065	427.255	P	687.346	5
5	102.055	556.298	E	590.293	4
6	101.071	684.356	Q	461.251	3
7	159.092	870.436	W	333.192	2
8	101.107		K	147.113	1

Figure S2 continued

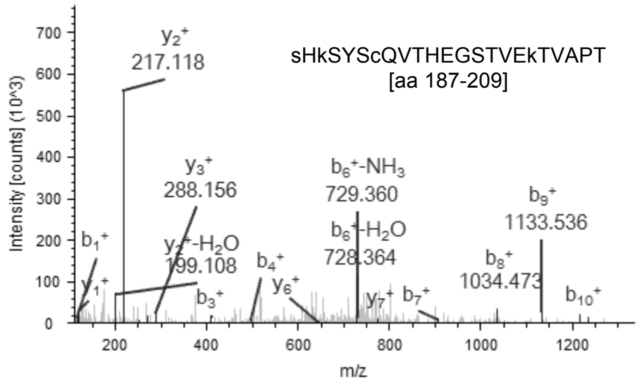

#1	Immonium	b⁺	Seq.	y⁺	#2
1	88.076	116.071	S-Dimethyl		23
2	110.071	253.130	H	2502.229	22
3	129.139	409.256	K-Dimethyl	2365.170	21
4	60.044	496.288	S	2209.044	20
5	136.076	659.351	Y	2122.012	19
6	60.044	746.383	S	1958.949	18
7	133.043	906.414	C-Carbamidomethyl	1871.917	17
8	101.071	1034.472	Q	1711.886	16
9	72.081	1133.541	V	1583.828	15
10	74.060	1234.588	T	1484.759	14
11	110.071	1371.647	H	1383.711	13
12	102.055	1500.690	E	1246.653	12
13	30.034	1557.711	G	1117.610	11
14	60.044	1644.743	S	1060.588	10
15	74.060	1745.791	T	973.556	9
16	72.081	1844.860	V	872.509	8
17	102.055	1973.902	E	773.440	7
18	129.139	2130.028	K-Dimethyl	644.398	6
19	74.060	2231.076	T	488.271	5
20	72.081	2330.145	V	387.224	4
21	44.049	2401.182	A	288.155	3
22	70.065	2498.234	P	217.118	2
23	74.060		T	120.066	1

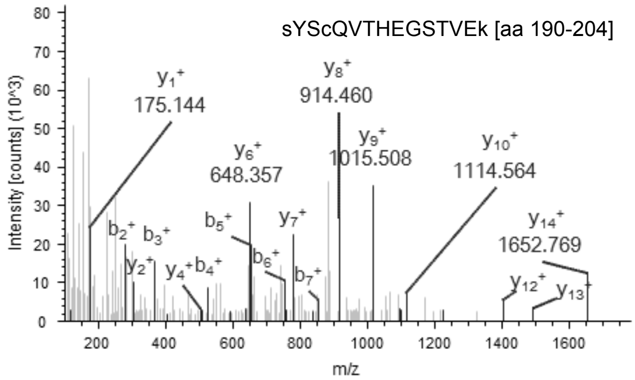

#1	Immonium	b⁺	Seq.	y⁺	#2
1	88.076	116.071	S-Dimethyl		15
2	136.076	279.134	Y	1652.758	14
3	60.044	366.166	S	1489.695	13
4	133.043	526.197	C-Carbamidomethyl	1402.663	12
5	101.071	654.255	Q	1242.632	11
6	72.081	753.324	V	1114.574	10
7	74.060	854.371	T	1015.505	9
8	110.071	991.430	H	914.458	8
9	102.055	1120.473	E	777.399	7
10	30.034	1177.494	G	648.356	6
11	60.044	1264.526	S	591.335	5
12	74.060	1365.574	T	504.303	4
13	72.081	1464.642	V	403.255	3
14	102.055	1593.685	E	304.187	2
15	129.139		K-Dimethyl	175.144	1

C

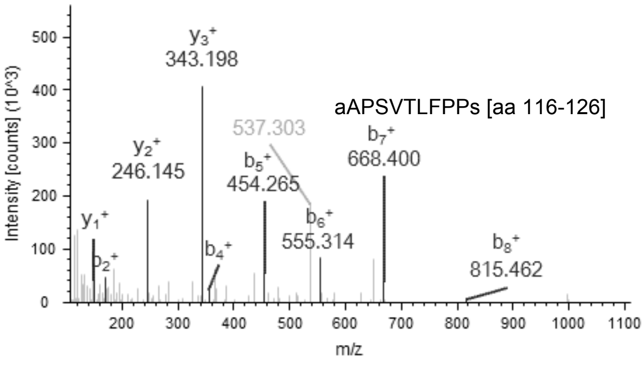

#1	Immonium	b⁺	Seq.	y⁺	#2
1	72.081	100.076	A-Dimethyl		11
2	44.049	171.113	A	1058.588	10
3	70.065	268.166	P	987.551	9
4	60.044	355.198	S	890.498	8
5	72.081	454.266	V	803.466	7
6	74.060	555.314	T	704.398	6
7	86.096	668.398	L	603.350	5
8	120.081	815.466	F	490.266	4
9	70.065	912.519	P	343.198	3
10	70.065	1009.572	P	246.145	2
11	103.087		S-Ethanolamine	149.092	1

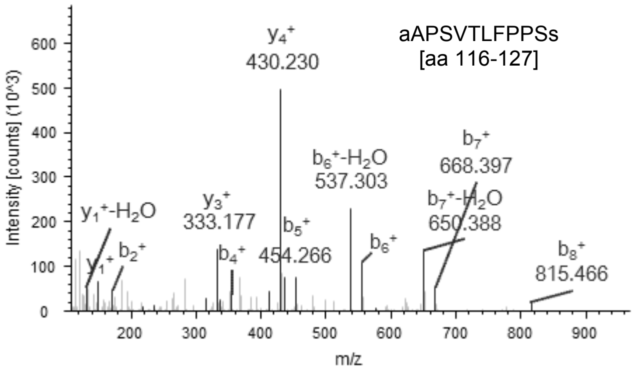

#1	Immonium	b⁺	Seq.	y⁺	#2
1	72.081	100.076	A-Dimethyl		12
2	44.049	171.113	A	1145.620	11
3	70.065	268.166	P	1074.583	10
4	60.044	355.198	S	977.530	9
5	72.081	454.266	V	890.498	8
6	74.060	555.314	T	791.430	7
7	86.096	668.398	L	690.382	6
8	120.081	815.466	F	577.298	5
9	70.065	912.519	P	430.230	4
10	70.065	1009.572	P	333.177	3
11	60.044	1096.604	S	236.124	2
12	103.087		S-Ethanolamine	149.092	1

Figure S2 continued

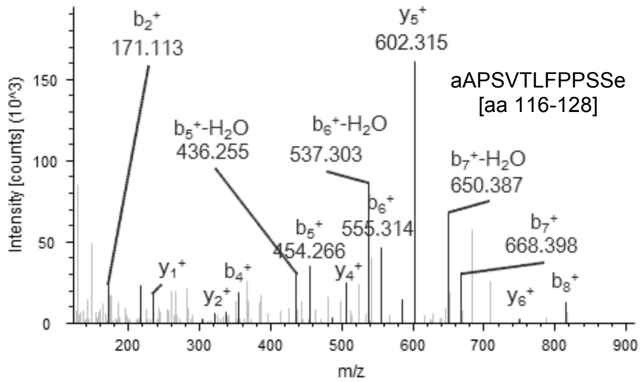

#1	Immonium	b+	Seq.	y+	#2
1	72.081	100.076	A-Dimethyl		13
2	44.049	171.113	A	1317.705	12
3	70.065	268.166	P	1246.668	11
4	60.044	355.198	S	1149.615	10
5	72.081	454.266	V	1062.583	9
6	74.060	555.314	T	963.515	8
7	86.096	668.398	L	862.467	7
8	120.081	815.466	F	749.383	6
9	70.065	912.519	P	602.314	5
10	70.065	1009.572	P	505.262	4
11	60.044	1096.604	S	408.209	3
12	60.044	1183.636	S	321.177	2
13	188.139		E-Ethanolamine-Ethanolamine	234.145	1

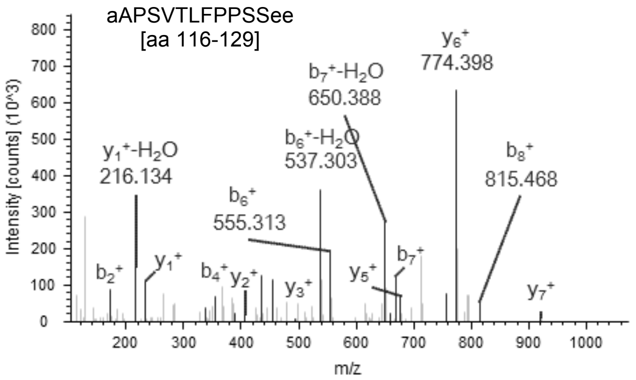

#1	Immonium	b+	Seq.	y+	#2
1	72.081	100.076	A-Dimethyl		14
2	44.049	171.113	A	1489.790	13
3	70.065	268.166	P	1418.753	12
4	60.044	355.198	S	1321.700	11
5	72.081	454.266	V	1234.668	10
6	74.060	555.314	T	1135.599	9
7	86.096	668.398	L	1034.552	8
8	120.081	815.466	F	921.468	7
9	70.065	912.519	P	774.399	6
10	70.065	1009.572	P	677.346	5
11	60.044	1096.604	S	580.294	4
12	60.044	1183.636	S	493.262	3
13	145.097	1355.721	E-Ethanolamine	406.230	2
14	188.139		E-Ethanolamine-Ethanolamine	234.145	1

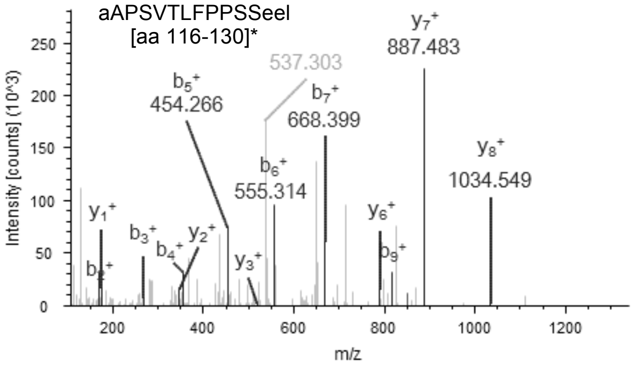

#1	Immonium	b+	Seq.	y+	#2
1	72.081	100.076	A-Dimethyl		15
2	44.049	171.113	A	1602.874	14
3	70.065	268.166	P	1531.837	13
4	60.044	355.198	S	1434.784	12
5	72.081	454.266	V	1347.752	11
6	74.060	555.314	T	1248.683	10
7	86.096	668.398	L	1147.636	9
8	120.081	815.466	F	1034.552	8
9	70.065	912.519	P	887.483	7
10	70.065	1009.572	P	790.431	6
11	60.044	1096.604	S	693.378	5
12	60.044	1183.636	S	606.346	4
13	145.097	1355.721	E-Ethanolamine	519.314	3
14	145.097	1527.805	E-Ethanolamine	347.229	2
15	129.139		L-Ethanolamine	175.144	1

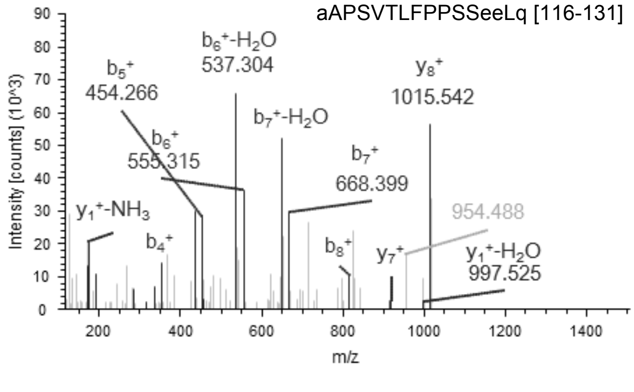

#1	Immonium	b+	Seq.	y+	#2
1	72.081	100.076	A-Dimethyl		16
2	44.049	171.113	A	1730.932	15
3	70.065	268.166	P	1659.895	14
4	60.044	355.198	S	1562.842	13
5	72.081	454.266	V	1475.810	12
6	74.060	555.314	T	1376.742	11
7	86.096	668.398	L	1275.694	10
8	120.081	815.466	F	1162.610	9
9	70.065	912.519	P	1015.542	8
10	70.065	1009.572	P	918.489	7
11	60.044	1096.604	S	821.436	6
12	60.044	1183.636	S	734.404	5
13	145.097	1355.721	E-Ethanolamine	647.372	4
14	145.097	1527.805	E-Ethanolamine	475.287	3
15	86.096	1640.889	L	303.203	2
16	144.113		Q-Ethanolamine	190.119	1

Figure S2 continued

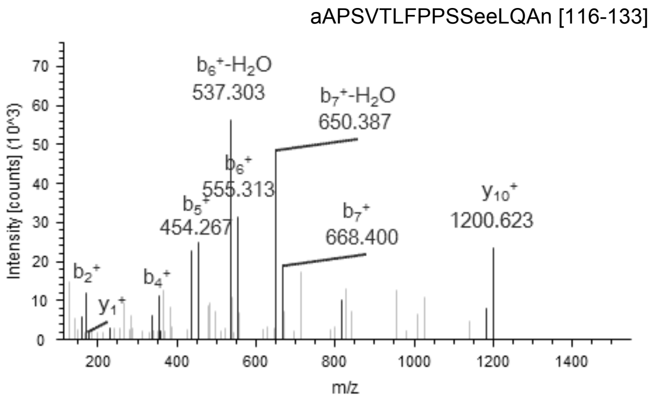

#1	Immonium	b+	Seq.	y+	#2
1	72.081	100.076	A-Dimethyl		18
2	44.049	171.113	A	1916.012	17
3	70.065	268.166	P	1844.975	16
4	60.044	355.198	S	1747.922	15
5	72.081	454.266	V	1660.890	14
6	74.060	555.314	T	1561.822	13
7	86.096	668.398	L	1460.774	12
8	120.081	815.466	F	1347.690	11
9	70.065	912.519	P	1200.622	10
10	70.065	1009.572	P	1103.569	9
11	60.044	1096.604	S	1006.516	8
12	60.044	1183.636	S	919.484	7
13	145.097	1355.721	E-Ethanolamine	832.452	6
14	145.097	1527.805	E-Ethanolamine	660.368	5
15	86.096	1640.889	L	488.283	4
16	101.071	1768.948	Q	375.199	3
17	44.049	1839.985	A	247.140	2
18	130.097		N-Ethanolamine	176.103	1

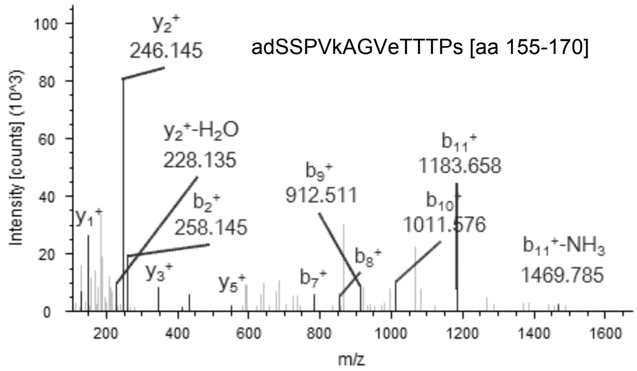

#1	Immonium	b+	Seq.	y+	#2
1	72.081	100.076	A-Dimethyl		16
2	131.082	258.145	D-Ethanolamine	1632.880	15
3	60.044	345.177	S	1474.811	14
4	60.044	432.209	S	1387.779	13
5	70.065	529.262	P	1300.747	12
6	72.081	628.330	V	1203.694	11
7	129.139	784.456	K-Dimethyl	1104.626	10
8	44.049	855.493	A	948.500	9
9	30.034	912.515	G	877.463	8
10	72.081	1011.583	V	820.441	7
11	145.097	1183.668	E-Ethanolamine	721.373	6
12	74.060	1284.716	T	549.288	5
13	74.060	1385.763	T	448.240	4
14	74.060	1486.811	T	347.193	3
15	70.065	1583.864	P	246.145	2
16	103.087		S-Ethanolamine	149.092	1

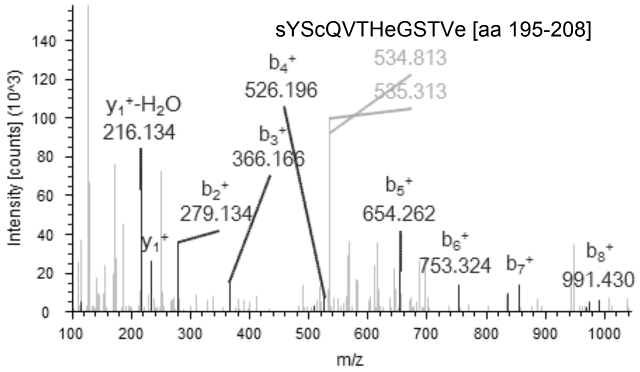

#1	Immonium	b+	Seq.	y+	#2
1	88.076	116.071	S-Dimethyl		14
2	136.076	279.134	Y	1625.759	13
3	60.044	366.166	S	1462.695	12
4	133.043	526.197	C-Carbamidomethyl	1375.663	11
5	101.071	654.255	Q	1215.633	10
6	72.081	753.324	V	1087.574	9
7	74.060	854.371	T	988.506	8
8	110.071	991.430	H	887.458	7
9	145.097	1163.515	E-Ethanolamine	750.399	6
10	30.034	1220.536	G	578.314	5
11	60.044	1307.568	S	521.293	4
12	74.060	1408.616	T	434.261	3
13	72.081	1507.685	V	333.213	2
14	188.139		E-Ethanolamine-Ethanolamine	234.145	1

D

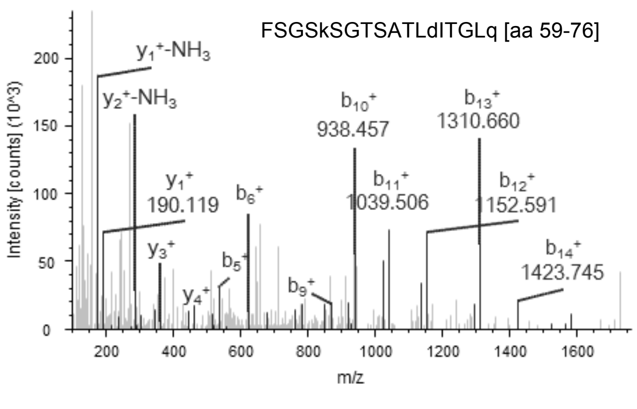

#1	Immonium	b+	Seq.	y+	#2
1	120.081	148.076	F		18
2	60.044	235.108	S	1736.939	17
3	30.034	292.129	G	1649.907	16
4	60.044	379.161	S	1592.885	15
5	129.139	535.287	K-Dimethyl	1505.853	14
6	60.044	622.320	S	1349.727	13
7	30.034	679.341	G	1262.695	12
8	74.060	780.389	T	1205.674	11
9	60.044	867.421	S	1104.626	10
10	44.049	938.458	A	1017.594	9
11	74.060	1039.505	T	946.557	8
12	86.096	1152.590	L	845.509	7
13	131.082	1310.659	D-Ethanolamine	732.425	6
14	86.096	1423.743	I	574.356	5
15	74.060	1524.790	T	461.272	4
16	30.034	1581.812	G	360.224	3
17	86.096	1694.896	L	303.203	2
18	144.113		Q-Ethanolamine	190.119	1

Figure S2 continued

aAPSVTLFPPSSee [aa 111-124]

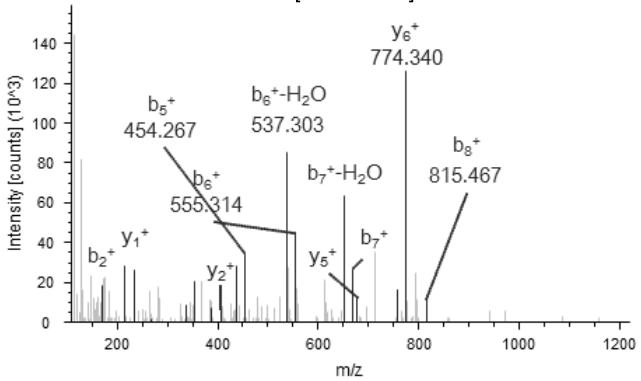

#1	Immonium	b⁺	Seq.	y⁺	#2
1	72.081	100.076	A-Dimethyl		14
2	44.049	171.113	A	1489.790	13
3	70.065	268.166	P	1418.753	12
4	60.044	355.198	S	1321.700	11
5	72.081	454.266	V	1234.668	10
6	74.060	555.314	T	1135.599	9
7	86.096	668.398	L	1034.552	8
8	120.081	815.466	F	921.468	7
9	70.065	912.519	P	774.399	6
10	70.065	1009.572	P	677.346	5
11	60.044	1096.604	S	580.294	4
12	60.044	1183.636	S	493.262	3
13	145.097	1355.721	E-Ethanolamine	406.230	2
14	188.139		E-Ethanolamine-Ethanolamine	234.145	1

aAPSVTLFPPSSeel [aa 111-125]*

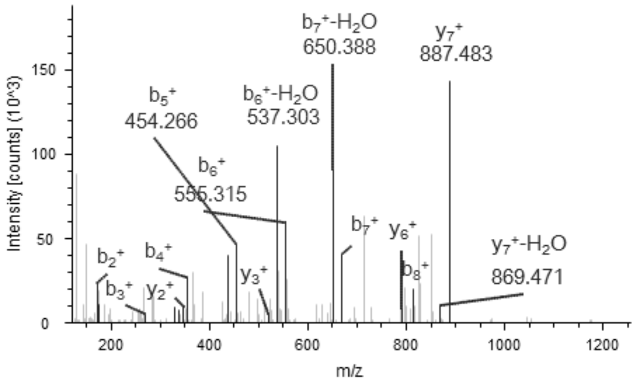

#1	Immonium	b⁺	Seq.	y⁺	#2
1	72.081	100.076	A-Dimethyl		15
2	44.049	171.113	A	1602.874	14
3	70.065	268.166	P	1531.837	13
4	60.044	355.198	S	1434.784	12
5	72.081	454.266	V	1347.752	11
6	74.060	555.314	T	1248.683	10
7	86.096	668.398	L	1147.636	9
8	120.081	815.466	F	1034.552	8
9	70.065	912.519	P	887.483	7
10	70.065	1009.572	P	790.431	6
11	60.044	1096.604	S	693.378	5
12	60.044	1183.636	S	606.346	4
13	145.097	1355.721	E-Ethanolamine	519.314	3
14	145.097	1527.805	E-Ethanolamine	347.229	2
15	129.139		L-Ethanolamine	175.144	1

aAPSVTLFPPSSeeLq [111-126]

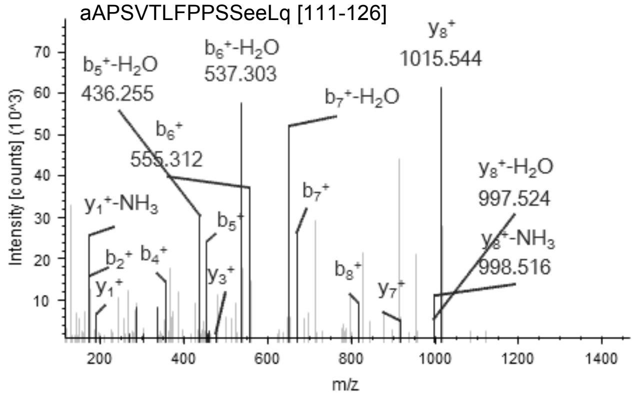

#1	Immonium	b⁺	Seq.	y⁺	#2
1	72.081	100.076	A-Dimethyl		16
2	44.049	171.113	A	1730.932	15
3	70.065	268.166	P	1659.895	14
4	60.044	355.198	S	1562.842	13
5	72.081	454.266	V	1475.810	12
6	74.060	555.314	T	1376.742	11
7	86.096	668.398	L	1275.694	10
8	120.081	815.466	F	1162.610	9
9	70.065	912.519	P	1015.542	8
10	70.065	1009.572	P	918.489	7
11	60.044	1096.604	S	821.436	6
12	60.044	1183.636	S	734.404	5
13	145.097	1355.721	E-Ethanolamine	647.372	4
14	145.097	1527.805	E-Ethanolamine	475.287	3
15	86.096	1640.889	L	303.203	2
16	144.113		Q-Ethanolamine	190.119	1

AAPSVTLFPPSSeeLQa [aa 111-127]

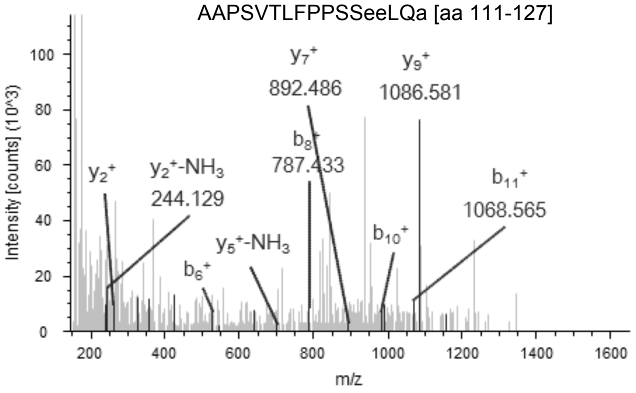

#1	Immonium	b⁺	Seq.	y⁺	#2
1	44.049	72.044	A		17
2	44.049	143.082	A	1801.969	16
3	70.065	240.134	P	1730.932	15
4	60.044	327.166	S	1633.880	14
5	72.081	426.235	V	1546.848	13
6	74.060	527.282	T	1447.779	12
7	86.096	640.366	L	1346.731	11
8	120.081	787.435	F	1233.647	10
9	70.065	884.488	P	1086.579	9
10	70.065	981.540	P	989.526	8
11	60.044	1068.572	S	892.473	7
12	60.044	1155.604	S	805.441	6
13	145.097	1327.689	E-Ethanolamine	718.409	5
14	145.097	1499.774	E-Ethanolamine	546.325	4
15	86.096	1612.858	L	374.240	3
16	101.071	1740.917	Q	261.156	2
17	87.092		A-Ethanolamine	133.097	1

Figure S2 continued

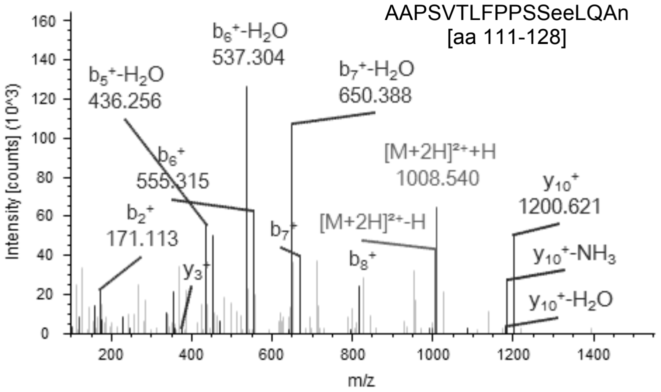

#1	Immonium	b+	Seq.	y+	#2
1	72.081	100.076	**A-Dimethyl**		18
2	44.049	171.113	**A**	1916.012	17
3	70.065	268.166	**P**	1844.975	16
4	60.044	355.198	**S**	1747.922	15
5	72.081	454.266	**V**	1660.890	14
6	74.060	555.314	**T**	1561.822	13
7	86.096	668.398	**L**	1460.774	12
8	120.081	815.466	**F**	1347.690	11
9	70.065	912.519	**P**	1200.622	10
10	70.065	1009.572	**P**	1103.569	9
11	60.044	1096.604	**S**	1006.516	8
12	60.044	1183.636	**S**	919.484	7
13	145.097	1355.721	**E-Ethanolamine**	832.452	6
14	145.097	1527.805	**E-Ethanolamine**	660.368	5
15	86.096	1640.889	**L**	488.283	4
16	101.071	1768.948	**Q**	375.199	3
17	44.049	1839.985	**A**	247.140	2
18	130.097		**N-Ethanolamine**	176.103	1

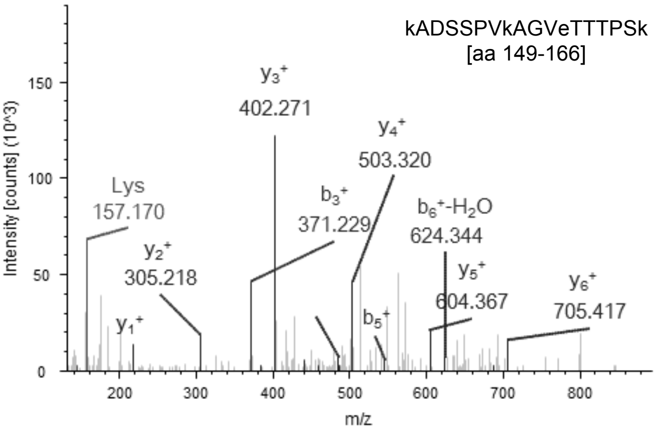

#1	Immonium	b+	Seq.	y+	#2
1	157.170	185.165	**K-Dimethyl-Dimethyl**		18
2	44.049	256.202	**A**	1817.001	17
3	88.039	371.229	**D**	1745.964	16
4	60.044	458.261	**S**	1630.937	15
5	60.044	545.293	**S**	1543.905	14
6	70.065	642.346	**P**	1456.873	13
7	72.081	741.414	**V**	1359.821	12
8	129.139	897.540	**K-Dimethyl**	1260.752	11
9	44.049	968.578	**A**	1104.626	10
10	30.034	1025.599	**G**	1033.589	9
11	72.081	1124.667	**V**	976.567	8
12	145.097	1296.752	**E-Ethanolamine**	877.499	7
13	74.060	1397.800	**T**	705.414	6
14	74.060	1498.848	**T**	604.366	5
15	74.060	1599.895	**T**	503.319	4
16	70.065	1696.948	**P**	402.271	3
17	60.044	1783.980	**S**	305.218	2
18	172.181		**K-Ethanolamine-Dimethyl**	218.186	1

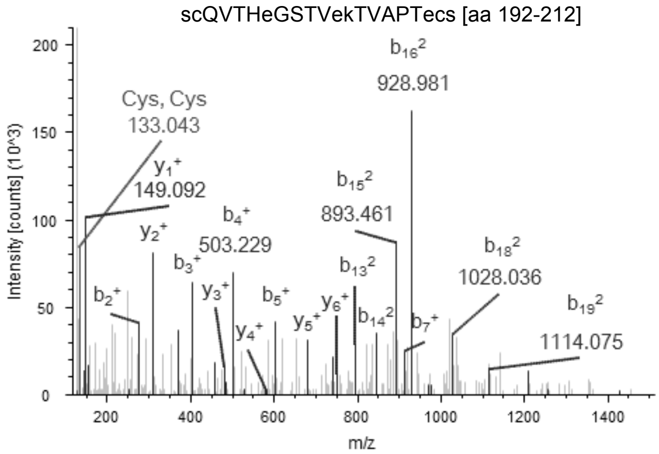

#1	Immonium	b+	Seq.	y+	#2
1	88.076	116.071	**S-Dimethyl**		21
2	133.043	276.101	**C-Carbamidomethyl**	2420.191	20
3	101.071	404.160	**Q**	2260.160	19
4	72.081	503.228	**V**	2132.102	18
5	74.060	604.276	**T**	2033.033	17
6	110.071	741.335	**H**	1931.986	16
7	145.097	913.420	**E-Ethanolamine**	1794.927	15
8	30.034	970.441	**G**	1622.842	14
9	60.044	1057.473	**S**	1565.820	13
10	74.060	1158.521	**T**	1478.788	12
11	72.081	1257.589	**V**	1377.741	11
12	145.097	1429.674	**E-Ethanolamine**	1278.672	10
13	129.139	1585.800	**K-Dimethyl**	1106.587	9
14	74.060	1686.848	**T**	950.461	8
15	72.081	1785.916	**V**	849.413	7
16	44.049	1856.953	**A**	750.345	6
17	70.065	1954.006	**P**	679.308	5
18	74.060	2055.054	**T**	582.255	4
19	145.097	2227.139	**E-Ethanolamine**	481.208	3
20	133.043	2387.169	**C-Carbamidomethyl**	309.123	2
21	103.087		**S-Ethanolamine**	149.092	1

* In peptide AAPSVTLFPPSSEEL (116-130 in AL-55 and 111-125 in AL-H7), which contains three consecutive C-terminal residues that can be labeled by EA (side chains of E 128 and E 129; C-terminus of L 130), incomplete labeling can translate into uncertainty regarding the labeled residues in all ions of the b and y series. Also the y1 ion is not diriment, since the ion with m/z 175.144 could correspond to either the EA-labeled L or to a dimethylated lysine from a co-eluting peptide. Therefore, in order to avoid mis-identification of labeled C-terminus, the labeling on L was attributed only upon detecting fragment ions indicating EA labeling on all the 3 C-terminal residues.

Figure S3. (A) Sequence homology between full length AL-55 and JTO (PDB 6MG4), and (B) between the variable domains of AL-H7 and that of PDB entry 6IC3

A

```
CLUSTAL 2.1 multiple sequence alignment


6MG4_B|PDBID|CHAIN|SEQUENCE      MNFMLNQPHSVSESPGKTVTISCTRSSGNIDSNYVQWYQQRPGSAPITVI
AL-55                            -NFMLTQPHSVSESPGKTLTISCTGSSASIASHYVQWYQQRPGGAPTTLI
                                  ****.************:***** **..* *:**********.** *:*

6MG4_B|PDBID|CHAIN|SEQUENCE      YEDNQRPSGVPDRFAGSIDRSSNSASLTISGLKTEDEADYYCQSYDAR-N
AL-55                            YENDQRPSEVPDRFSGSIDSSSNSASLTISGLKTEDEADYYCQSYDGNNH
                                 **::**** *****:**** ***************************.. :

6MG4_B|PDBID|CHAIN|SEQUENCE      VVFGGGTRLTVLGQPKAAPSVTLFPPSSEELQANKATLVCLISDFYPGAV
AL-55                            WVFGGGTKLTVLSQPKAAPSVTLFPPSSEELQANKATLVCLISDFYPGAV
                                  ******:****.**************************************

6MG4_B|PDBID|CHAIN|SEQUENCE      TVAWKADSSPVKAGVETTTPSKQSNNKYAASSYLSLTPEQWKSHKSYSCQ
AL-55                            TVAWKADSSPVKAGVETTTPSKQSNNKYAASSYLSLTPEQWKSHKSYSCQ
                                 **************************************************

6MG4_B|PDBID|CHAIN|SEQUENCE      VTHEGSTVEKTVAPTECS
AL-55                            VTHEGSTVEKTVAPTECS
                                 ******************
```

B

```
CLUSTAL 2.1 multiple sequence alignment


6IC3_A|PDBID|CHAIN|SEQUENCE      --VLTQPPSASGTPGQRVTISCSGRSSNIGRNLVKWYQQFPGTAPKLLIY
AL-H7|V-domain                   QSVLTQPPSVSAAPGQKVTISCS----NVGKNFVSWYQQFPGTAPKVVIY
                                   *******.*.:***:******    *:*:*:*.***********::**

6IC3_A|PDBID|CHAIN|SEQUENCE      SNDQRPSGVPDRFSGSKSGTSASLAVSGLQSEDEADYYCAAWDATLNAWV
AL-H7|V-domain                   DTDKRPSDIPDRFSGSKSGTSATLDITGLQTGDEADYYCGTWDSGLNGGV
                                 ..*:***.:*************:* ::***: *******.:**: **. *

6IC3_A|PDBID|CHAIN|SEQUENCE      FGGGTKLTVLSQPKAAPS
AL-H7|V-domain                   FGGGTKVTVLGQPKAAPS
                                 ******:***.*******
```

Figure S4. Absolute frequency of the amino acids involved in the cleaved bonds identified in the study (blue graph: amino acids located at the N-terminal side of the bond; red graph: amino acids located at the C-terminal side of the bond).

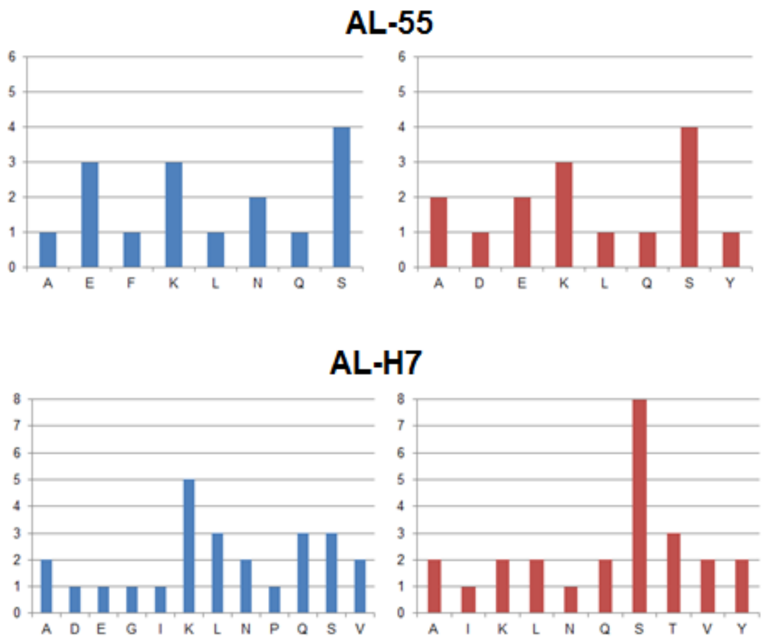

Figure S5. Gel image corresponding to panel A in Figure 2 of the main text, displaying the original molecular weight marker lane.

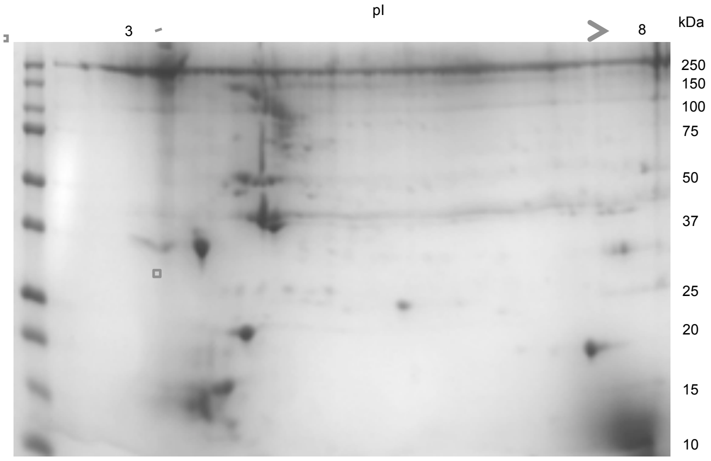

Figure S6. Details (boxed regions) of the coomassie-stained 2D-PAGE gels shown in Figure 2 of the text. The spots belonging to the same trains were pooled, subjected to in-gel digestion and peptide extraction, and analyzed by LC-MS/MS. *Only the semitryptic peptides ending with positions corresponding to those derivatized in the terminomics study were considered as bona fide terminal peptides of the LC fragments, and only these N- or C-terminal residues were listed in the table. Most of the peptides containing termini labeled in solution, however, were missing in the gel-based analysis, and in some instances none of the labeled N- or C-terminal peptides were detectable. The right column of the table shows, for each train of spots, the regions of sequence covered by the identified peptides (green) and the N- and C-termini detected. **Spot 9 of AL-55, whose MW is not compatible with the full length LC, is broad and scarcely defined. The fact that both N-terminal and C-terminal peptides were found in this spot likely reflects the presence of co-migrating fragments, consisting of either the N-terminal part, or the C-terminal part of the LC.

AL-H7

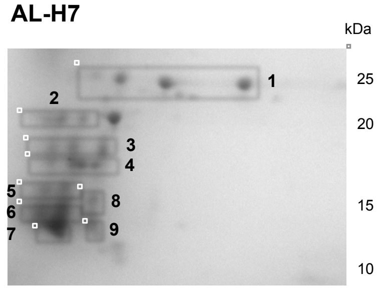

Spot #	N-termini	C-termini	Sequence coverage
1	1, 3, 4	Not identified	
2	Not identified	Not identified	
3	2, 3, 4, 5	Not identified	
4	2, 3, 4, 5	Not identified	
5	2, 4, 5,	Not identified	
6	2, 3, 4, 5	124, 126, 127	
7	2, 3, 4, 5	128	
8	4, 5	Not identified	
9	2, 3, 4, 5	Not identified	

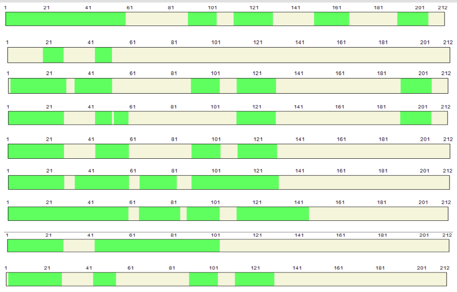

AL-55

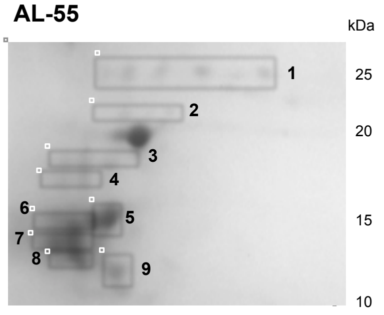

Spot #	N-termini	C-termini	Sequence coverage
1	Not identified	Not identified	
2	177	Not identified	
3	Not identified	Not identified	
4	Not identified	Not identified	
5	Not identified	Not identified	
6	64	128, 129	
7	1, 64, 135	128, 129	
8	1, 64	128, 129	
9	64	128, 129	

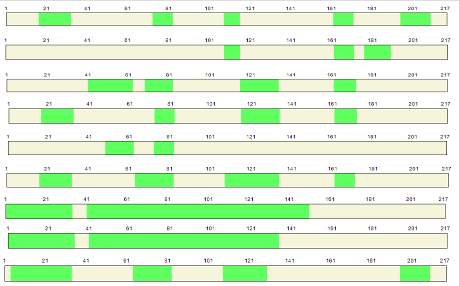

Table S1. Annotation of the cleavage sites on the native and fibrillar light chains' structures. * numbering in parenthesis refers to positions indicated in the PDB file structure, due to the non-canonical numbering adopted.

Native JTO (6MG4)*	Amyloid AL-55 (6HUD)	Native H7 (5MUH)	Amyloid λ1 (6IC3)
**VL**		**VL**	
D53 (D51) Exposed loop (CDR2)	No proteolytic sites in the fibrillar core	S2 Flexible in the structure	
A65 (A63) β strand		V3 Flexible in the structure	
		L4 β strand	
		T5 β strand	
		S9 β strand	
		V10 β strand	
		T39 Partially exposed loop	T43 β strand
		I45 Hydrophobic core	I49 β strand
		Q76 Exposed loop	Q80 β strand and buried
**CL**		**CL**	
127-131 (121-126) α helix		124-129 α helix	
N133 (N128) Exposed loop		A130 β strand	
A135 (A130) β strand and buried		S137 β strand and buried	
S170 (S165) β strand and buried		S152 Exposed loop	
Q172 (Q167) β strand		K166 β strand	
Y177 (Y173) β strand and buried		Q167 β strand	
S180 (S176) β strand and buried		K171 Loop	
S181 (S177) β strand and buried		Y172 β strand and buried	
S192 (S188) Exposed loop		Y177 β strand and buried	
E208 (E206) β strand		S179 β strand	
C-*terminus*		S187 Exposed loop	
		C-*terminus*	

Table S2. List of all identified labeled peptides in AL-55 and AL-H7 LCs.

Positions	Sequence	Modifications	Charge	m/z [Da]	XCorr
AL-55 [135-148]	ATLVCLISDFYPGA	1×Dimethyl [N-Term]; 1×Carbamidomethyl [C5]	2	777.899	2.54
AL-55 [53-62]	DQRPSEVPDR	1×Dimethyl [N-Term]	3	409.542	2.88
AL-55 [1-16]	NFMLTQPHSVSESPGK	1×Dimethyl [N-Term]	2	893.944	4.57
AL-55 [172-191]	QSNNKYAASSYLSLTPEQWK	1×Dimethyl [N-Term]	3	781.726	4.39
AL-55 [64-82]	SGSIDSSSNSASLTISGLK	1×Dimethyl [N-Term]	2	919.969	6.21
AL-55 [180-191]	SSYLSLTPEQWK	1×Dimethyl [N-Term]	2	733.880	4.51
AL-55 [181-191]	SYLSLTPEQWK	1×Dimethyl [N-Term]	2	690.368	2.58
AL-55 [177-191]	YAASSYLSLTPEQWK	1×Dimethyl [N-Term]	2	886.450	4.08
AL-H7 [130-144]	ATLVCLISDFYPGAV	1×Dimethyl [N-Term]; 1×Carbamidomethyl [C5]	2	827.435	2.99
AL-H7 [45-58]	IYDTDKRPSDIPDR	2×Dimethyl [N-term; K6]	3	582.971	2.39
AL-H7 [129-149]	KATLVCLISDFYPGAVTVAWK	2×Dimethyl [N-Term; K1]; 1×Carbamidomethyl [C6]	3	799.112	5.89
AL-H7 [171-186]	KYAASSYLSLTPEQWK	2×Dimethyl [N-term; K1]	2	964.513	3.73
AL-H7 [4-17]	LTQPPSVSAAPGQK	1×Dimethyl [N-Term]	2	704.893	2.72
AL-H7 [167-186]	QSNNKYAASSYLSLTPEQWK	1×Dimethyl [N-Term]	3	781.724	5.33
AL-H7 [137-149]	SDFYPGAVTVAWK	1×Dimethyl [N-Term]	2	734.876	2.61
AL-H7 [187-209]	SHKSYSCQVTHEGSTVEKTVAPT	3×Dimethyl [N-Term; K3; K18]; 1×Carbamidomethyl [C7]	4	655.079	6.44
AL-H7 [179-186]	SLTPEQWK	1×Dimethyl [N-Term]	2	508.775	2.21
AL-H7 [152-166]	SSPVKAGVETTTPSK	2×Dimethyl [N-Term; K5]	3	515.622	2.97
AL-H7 [175-186]	SSYLSLTPEQWK	1×Dimethyl [N-Term]	2	733.879	3.53
AL-H7 [2-17]	SVLTQPPSVSAAPGQK	1×Dimethyl [N-Term]	3	532.2970	4.41
AL-H7 [9-27]	SVSAAPGQKVTISCSNVGK	2×Dimethyl [N-Term; K9]; 1×Carbamidomethyl [C14]	3	649.351	6.05
AL-H7 [190-204]	SYSCQVTHEGSTVEK	2×Dimethyl [N-Term; K15]; 1×Carbamidomethyl [C4]	2	884.415	3.54
AL-H7 [39-58]	TAPKVVIYDTDKRPSDIPDR	3×Dimethyl [N-Term; K4; K12]	4	593.332	5.12
AL-H7 [5-17]	TQPPSVSAAPGQK	1×Dimethyl [N-Term]	2	648.352	3.93
AL-H7 [3-17]	VLTQPPSVSAAPGQK	1×Dimethyl [N-Term]	3	503.288	3.83
AL-H7 [10-27]	VSAAPGQKVTISCSNVGK	2×Dimethyl [N-Term; K8]; 1×Carbamidomethyl [C13]	3	620.341	3.15
AL-H7 [172-185]	YAASSYLSLTPEQW	1×Dimethyl [N-Term]	2	822.401	2.89
AL-H7 [177-189]	YLSLTPEQWKSHK	2×Dimethyl [N-Term; K]	4	418.982	3.33

AL-55 [116-126]	AAPSVTLFPPS	1×Dimethyl [N-Term]; 1×Ethanolamine [C-Term]	2	579.332	2.16
AL-55 [116-127]	AAPSVTLFPPSS	1×Dimethyl [N-Term]; 1×Ethanolamine [C-Term]	2	622.848	2.36
AL-55 [116-128]	AAPSVTLFPPSSE	1×Dimethyl [N-Term]; 2×Ethanolamine [C-Term; E]	2	708.889	2.31
AL-55 [116-129]	AAPSVTLFPPSSEE	1×Dimethyl [N-Term]; 3×Ethanolamine [E13; C-Term; E]	2	794.932	2.39
AL-55 [116-130]	AAPSVTLFPPSSEEL	1×Dimethyl [N-Term]; 3×Ethanolamine [E13; E14; C-Term]	2	851.474	2.41
AL-55 [116-131]	AAPSVTLFPPSSEELQ	1×Dimethyl [N-Term]; 3×Ethanolamine [E13; E14; C-Term]	2	915.501	2.36
AL-55 [116-133]	AAPSVTLFPPSSEELQAN	1×Dimethyl [N-Term]; 3×Ethanolamine [E13; E14; C-Term]	2	1008.043	2.3
AL-55 [155-170]	ADSSPVKAGVETTTPS	2×Dimethyl [N-Term; K7]; 3×Ethanolamine [D2; E11; C-Term]	2	866.477	2.18
AL-55 [195-208]	SYSCQVTHEGSTVE	1×Dimethyl [N-Term]; 3×Ethanolamine [E9; C-Term; E]; 1×Carbamidomethyl [C4]	3	580.944	2.32
AL-H7 [111-124]	AAPSVTLFPPSSEE	1×Dimethyl [N-Term]; 3×Ethanolamine [E13; C-Term; E]	2	794.932	2.31
AL-H7 [111-125]	AAPSVTLFPPSSEEL	1×Dimethyl [N-Term]; 3×Ethanolamine [E13; E14; C-Term]	2	851.477	2.64
AL-H7 [111-126]	AAPSVTLFPPSSEELQ	1×Dimethyl [N-Term]; 3×Ethanolamine [E13; E14; C-Term]	2	915.500	2.47
AL-H7 [111-127]	AAPSVTLFPPSSEELQA	3×Ethanolamine [E13; E14; C-Term]	2	937.0100	2.55
AL-H7 [111-128]	AAPSVTLFPPSSEELQAN	1×Dimethyl [N-Term]; 3×Ethanolamine [E13; E14; C-Term]	2	1008.045	2.3
AL-H7 [59-76]	FSGSKSGTSATLDITGLQ	2×Ethanolamine [D13; C-Term]; 1×Dimethyl [K]	2	942.511	3.71
AL-H7 [149-166]	KADSSPVKAGVETTTPSK	4×Dimethyl [N-Term; K1; K8; K18]; 2×Ethanolamine [E12; C-Term]	4	501.045	3.94
AL-H7 [192-212]	SCQVTHEGSTVEKTVAPTECS	2×Dimethyl [N-Term; K13]; 4×Ethanolamine [E7; E12; E19; C-Term]; 2×Carbamidomethyl [C2; C20]	3	845.757	5.22
